# Common and Distinctive Intercellular Communication Patterns in Human Obstructive and Nonobstructive Hypertrophic Cardiomyopathy

**DOI:** 10.3390/ijms23020946

**Published:** 2022-01-15

**Authors:** Christina J. Codden, Michael T. Chin

**Affiliations:** 1 Molecular Cardiology Research Institute, Tufts Medical Center, Boston, MA 02111, USA; cjcodden@gmail.com; 2 Tufts Hypertrophic Cardiomyopathy Center and Research Institute, Boston, MA 02111, USA

**Keywords:** Hypertrophic Cardiomyopathy, left ventricular outflow tract obstruction, single-nucleus RNA-sequencing, dendritic cells, integrin-β1

## Abstract

Hypertrophic Cardiomyopathy (HCM) is a common inherited disorder characterized by unexplained left ventricular hypertrophy with or without left ventricular outflow tract (LVOT) obstruction. Single-nuclei RNA-sequencing (snRNA-seq) of both obstructive and nonobstructive HCM patient samples has revealed alterations in communication between various cell types, but no direct and integrated comparison between the two HCM phenotypes has been reported. We performed a bioinformatic analysis of HCM snRNA-seq datasets from obstructive and nonobstructive patient samples to identify differentially expressed genes and distinctive patterns of intercellular communication. Differential gene expression analysis revealed 37 differentially expressed genes, predominantly in cardiomyocytes but also in other cell types, relevant to aging, muscle contraction, cell motility, and the extracellular matrix. Intercellular communication was generally reduced in HCM, affecting the extracellular matrix, growth factor binding, integrin binding, PDGF binding, and SMAD binding, but with increases in adenylate cyclase binding, calcium channel inhibitor activity, and serine-threonine kinase activity in nonobstructive HCM. Increases in neuron to leukocyte and dendritic cell communication, in fibroblast to leukocyte and dendritic cell communication, and in endothelial cell communication to other cell types, largely through changes in the expression of integrin-β1 and its cognate ligands, were also noted. These findings indicate both common and distinct physiological mechanisms affecting the pathogenesis of obstructive and nonobstructive HCM and provide opportunities for the personalized management of different HCM phenotypes.

## 1. Introduction

Hypertrophic Cardiomyopathy (HCM) is a common inherited disorder affecting approximately 1 out of 500 live births that is characterized by unexplained left ventricular hypertrophy. In most cases, the hypertrophy is asymmetric, involving the interventricular septum (IVS), and can lead to left ventricular outflow (LVOT) obstruction, an important cause of heart failure symptoms. Patients with LVOT obstruction often do well with septal reduction therapy, which is achieved by surgical myectomy in most cases or by alcohol septal ablation for high-risk surgical candidates [1]. A significant percentage (30%) of patients do not develop LVOT obstruction but can develop intractable heart failure, despite guideline directed medical management, and often proceed to heart transplantation. The factors that influence the development of an obstructive phenotype vs. a nonobstructive phenotype are unknown.

HCM has traditionally been considered a disease of the sarcomere, based on numerous familial cohorts with mutations in sarcomere genes [2]. Broader analysis of the general HCM population has led to the consensus that up to 70% of HCM patients do not have demonstrable sarcomere gene mutations, leading some to call for new paradigms to understand the origins of HCM [3]. The decision to pursue routine genetic screening must thus be individualized [4], and the consideration of mechanisms beyond the sarcomere has been discussed [5]. The comparison of patients with sarcomere mutations to those who do not has indicated a higher incidence of adverse events in those with known sarcomere mutations [6]. More recently, large-scale genetic analyses have identified polygenic contributors to HCM that can act as modifiers of existing sarcomere mutations and also affect modifiable traits such as diastolic blood pressure [7,8,9]. Further genotype-phenotype correlations have been elusive, however, and, to the best of our knowledge, no genetic features distinguish obstructive HCM and nonobstructive HCM.

Single-cell RNA-sequencing and single-nuclei RNA-sequencing (snRNA-seq) have facilitated the analysis of cellular diversity and intercellular communication in the human heart [10,11,12,13]. We have previously defined the cellular diversity of the human heart IVS [12] and delineated important changes in communication between the cells of the IVS in obstructive HCM through mechanisms involving integrin-β1 (ITGB1) and the extracellular matrix (ECM) [13]. We have also performed a separate study comparing the snRNA-seq profiles of nonobstructive HCM IVS tissue with normal IVS tissue and found an increase in dendritic cell communication [14]. To date, no direct comparisons of single-cell gene expression patterns in obstructive and nonobstructive HCM exist. Here, we report a bioinformatics analysis of snRNA-seq datasets from obstructive and nonobstructive HCM to identify common and distinct pathological pathways that may permit therapeutic targeting. Both types of HCM have revealed a general decrease in ligand–receptor interactions involving integrin-β1 and its cognate extracellular matrix ligands, but the overall decrease was larger in magnitude among obstructive HCM samples. Nonobstructive HCM was also notable for its increased calcium channel activity, adenylate cyclase binding, and serine-threonine kinase activator activity signaling, thereby implicating signaling pathways that may be specific to nonobstructive HCM. Distinctive features of each type of HCM, both obstructive and nonobstructive, may thus guide precision medicine approaches to the treatment of each condition.

## 2. Results

### 2.1. Integration, Clustering, and Cell Assignment of snRNA-seq Datasets from Obstructive HCM, Nonobstructive HCM, and Unused Organ Donor Heart Tissue

**Figure 1:**
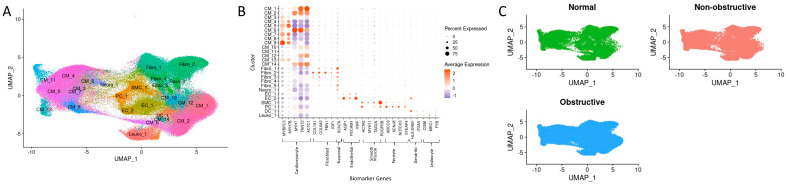
SnRNA-seq cluster identification, biomarker gene expression, and nuclei distribution across conditions. (**A**) UMAP representation of clusters with cell assignment labels. (**B**) Dot plot representation of cell type specific marker genes used to assign cell identity to each cluster. (**C**) UMAP representation of clusters visualized according to disease label.

The 22 datasets of control (6), obstructive HCM (10), and nonobstructive HCM (6) samples have been previously described [12,13,14]. The datasets are available from the Gene Expression Omnibus (GEO) database under accession numbers GSE161921, GSE174691, and GSE181764. Sn-RNAseq data from all 22 samples were combined into one dataset using the Seurat Integration function [15]. The final dataset consisted of 264481 nuclei, with 181113 nuclei from obstructive HCM hearts, 49010 nuclei from nonobstructive HCM hearts, and 34358 nuclei from organ donor hearts. The clustering of the integrated dataset initially revealed 28 cell populations. Two clusters were small, with one cluster consisting of 142 nuclei derived from a single sample and another cluster consisting of 82 nuclei, 79 of which were derived from the same single sample. These clusters were removed from further analysis by setting a cutoff point of 200 nuclei per cluster. The final dataset thus consisted of 26 clusters. The cell-type assignment for each cluster was performed as described previously [12,13,14]. Briefly, clusters were assessed for the expression of cell type-specific gene markers, with differentially expressed genes queried against panglaoDB [16], gene ontology (GO) carried out using GOStats [17], and with the use of Ingenuity Pathway Analysis [18]. Clusters were assigned a cell type if there was consensus between at least two methods. A Uniform Manifold Approximation and Projection (UMAP) plot with assigned cell types and an accompanying dot plot showing marker gene expression are shown in Figure 1A,B. Of the 26 clusters, 14 were assigned as cardiomyocytes, 5 were assigned as fibroblasts, and 2 were assigned as endothelial cells, as well as single clusters of neurons, smooth muscle cells, pericytes, dendritic cells, and leukocytes, for a total of 8 unique cell types. Cardiomyocyte and fibroblast diversity in the human heart likely reflects cell subtypes in different physiological states and has been previously reported in multiple studies [10,11,12,13,14]. No clusters specific to a single condition were observed (Figure 1C).

### 2.2. Trajectory Analysis and Differential Gene Expression Reveals Differences in Cell-Specific Gene Expression between Nonobstructive and Obstructive HCM

**Figure 2:**
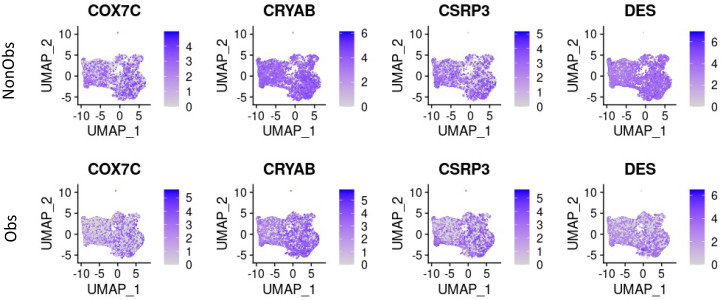
Differential expression of four representative cardiomyocyte genes in UMAP space from Table 1, revealing increased expression in nonobstructive HCM cardiomyocytes.

To determine the relationships between the cell types represented by nuclei in different clusters and between cells represented by nuclei within a cluster, we performed trajectory analysis using Monocle3 [19]. The previous analysis of relationships between cellular subtypes in obstructive HCM and normal tissue [13] and between cellular subtypes in nonobstructive HCM and normal tissue [14] by trajectory analysis did not reveal any significant differences between each type of HCM and normal tissue trajectories, although many genes were differentially expressed between conditions along the trajectories. Similarly, in this work no differences were seen in trajectories between obstructive and nonobstructive HCM (data not shown), although differences were seen in gene expression along the trajectory paths. Differentially expressed genes between nonobstructive and obstructive HCM were identified using spatial autocorrelation, filtered by a Moran value > 0.1 if a value was only present in one condition or an absolute difference > 0.1 if a Moran value was present for a gene in both conditions, as has been previously described for obstructive HCM compared to normal tissue [13] and nonobstructive HCM compared to normal tissue [14]. A total of 116 genes were identified using this filter, and are listed in Supplementary Table S1, along with the relevant cell type(s). Of these 116 genes, 37 were noted to be of visual interest based upon a comparative expression in UMAP space (Table 1). Many of the differentially expressed genes were manifested in cardiomyocytes. Only one of these genes, CSRP3, has been definitively linked to HCM [20], while another, CRYAB, has been reported [21]. The differential expression of four representative cardiomyocyte genes (COX7C, CRYAB, CSRP3, and DES) in UMAP space is shown in Figure 2.

**Figure 3:**
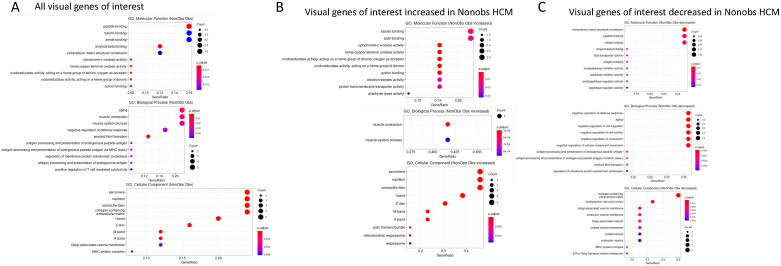
Gene ontology analysis of genes differentially expressed in nonobstructive and obstructive HCM. (**A**) Analysis of molecular functions, biological processes, and cellular components showing significant changes in nonobstructive compared to obstructive HCM. (**B**) GO classifications of genes that are increased in nonobstructive HCM compared to obstructive HCM. (**C**) GO classifications of genes that are decreased in nonobstructive HCM compared to obstructive HCM.

To determine the molecular functions, biological processes, and cellular components associated with differentially expressed genes, we performed a GO analysis. Differentially expressed genes were associated with molecular functions including peptide binding, tubulin binding, and amide binding. Associated biological processes included aging, muscle contraction, and muscle system processes. The associated cellular components included the sarcomere, myofibrils, contractile fibers, and collagen-containing extracellular matrix (Figure 3A). The GO analysis of genes that specifically showed an increased expression in nonobstructive HCM revealed an association with molecular functions such as tubulin binding, actin binding, and various oxidase and oxidoreductase activities. GO analysis also showed an increase in the biological process of muscle contraction and the cellular components sarcomere, myofibrils, and contractile fibers in nonobstructive HCM (Figure 3B). The GO analysis of genes that specifically show a reduced expression in nonobstructive HCM revealed an association with molecular functions such as extracellular matrix structural constituents; peptide binding; amide binding; and biological processes such as the negative regulation of the virus defense response, aging, the negative regulation of cell migration, the negative regulation of cell motility, the negative regulation of locomotion, the negative regulation of cellular component movement, and the cellular component collagen containing extracellular matrix (Figure 3C).

### 2.3. Ligand–Receptor Pair Gene Expression Analysis Reveals Alterations in Ligand and Receptor Gene Expression That Varies by Cell Type in Nonobstructive and Obstructive HCM

**Figure 4:**
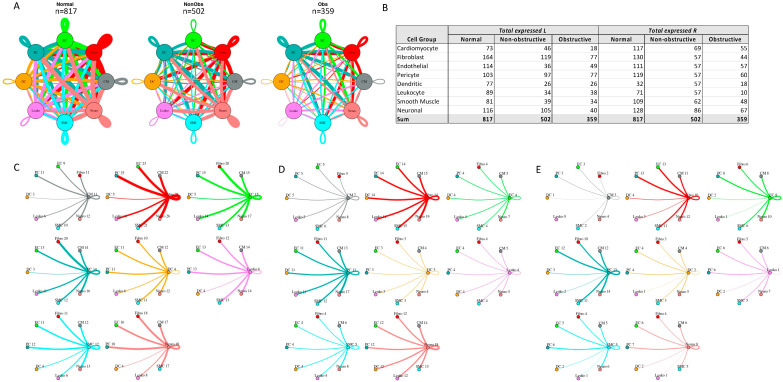
Intercellular communication networks in normal, nonobstructive, and obstructive HCM. (**A**) Cell–cell communication networks between cardiac cell types in normal control (left), nonobstructive (middle), and obstructive HCM (right) conditions. Line color indicates ligand broadcasting by the cell population with the same color. Lines connect to cell types which expressed cognate receptors. Line thickness is proportional to the number of uniquely expressed ligand–receptor pairs. Loops indicate communication within a cell type. (**B**) Quantity of ligands and receptors in expressed ligand–receptor pairs described by cell type and condition (normal control, nonobstructive HCM, or obstructive HCM). (**C**–**E**) Cell–cell communication networks broken down by cell type in normal control (**C**), nonobstructive HCM (**D**), and obstructive HCM (**E**) conditions. Figure formatting in C-E follows panel A and numbers indicate the quantity of uniquely expressed ligand–receptor pairs between the broadcasting cell type (expressing ligand) and receiving cell type (expressing receptor).

To determine the relative density of potential intercellular interactions in normal, nonobstructive HCM, and obstructive HCM IVS tissue, we analyzed ligand–receptor pair gene expression in the single-nuclei datasets derived from each tissue, as previously described [13,14,22]. We examined the expression of 3627 unique human ligand–receptor (L–R) pairs derived from combining a curated set of 2557 human L–R pairs [23] with another set of 3398 human L–R pairs [24] and eliminating duplicates, as we have done previously [14]. L–R components were considered expressed if expression was detectable in more than 20% of the cells within a cell type, as has been done in prior studies [13,14,22,25]. Among the different conditions, normal cells demonstrated the greatest number of potential L–R interactions among the 8 cell types (n = 817), compared to nonobstructive HCM cells (n = 502) and obstructive HCM cells (n = 359) (Figure 4A). Among cell types, fibroblasts broadcasted the most ligands among all cell types in all conditions (Figure 4B). In obstructive HCM when compared to nonobstructive HCM, a reduction in ligand expression was particularly notable in cardiomyocytes, fibroblasts, pericytes, and neurons (Figure 4B). In nonobstructive HCM, endothelial cells showed a disproportionate decrease in ligand expression compared to obstructive HCM, while reductions in dendritic cell, leukocyte, and smooth muscle cell ligands were comparable in both types of HCM (Figure 4B). Receptor expression was disproportionally reduced in most cell types in obstructive HCM compared to nonobstructive HCM, including cardiomyocytes, fibroblasts, dendritic cells, leukocytes, smooth muscle cells, and neurons, but comparable in endothelial cells and pericytes (Figure 4B).

The greatest potential relative reductions in intercellular communication in obstructive HCM compared to nonobstructive HCM involved pathways between neurons and fibroblasts (12 total L–R pairs vs. 4 total L–R pairs, fibroblasts and leukocytes (14 L–R pairs vs. 3 L–R pairs), fibroblasts and dendritic cells (14 L–R pairs vs. 4 L–R pairs), and neurons and dendritic cells (12 L–R pairs vs. 2 L–R pairs; Figure 4C–E, Supplementary Figure S1). The reduction in neuron to fibroblast communication was due to the loss of neuronal expression of several cognate ligands for the ITGB1 receptor (COL1A2, COL4A1, COL6A1, FN1, LAMA2, TGM2) and loss of RYR2 expression in fibroblasts. The reduction in fibroblast to leukocyte communication is attributable to the loss of ITGB1 receptor in leukocytes, which disabled communications with several cognate ligands (COL1A2, COL3A1, COL4A1, COL6A1, COL6A2, COL6A3, FN1, LAMA2, LGALS1, LUM). The reduction in fibroblast to dendritic cell communication is again attributable to the loss of ITGB1 receptor expression in dendritic cells, which disrupted communication with the same set of cognate ligands described for fibroblast to leukocyte communication. The reduction in neuron to dendritic cell communication was due primarily to the loss of ITGB1 receptor expression in dendritic cells but, as noted, there was also a decrease in the ITGB1 ligand expression in neurons. It is important to view these changes in a more global comparison with intercellular communication in normal tissues, where reductions in neuron to fibroblast communication are seen in both types of HCM but more severe in obstructive HCM, while a reduction in fibroblast to leukocyte communication is only observed in obstructive HCM compared to normal tissue. The relative reduction in fibroblast and neuron to dendritic cell communication in obstructive HCM is a consequence of the increased communication between these cell types in nonobstructive HCM rather than a large change compared to normal tissue (Figure 4C–E), consistent with the enhanced role of dendritic cells in the pathogenesis of nonobstructive HCM, as we have reported previously [14]. Dendritic cells play an important role in antigen presentation and immune system activation and have been implicated in the pathogenesis of heart failure [26,27]. Integrin-b1 is known to affect dendritic cell anti-inflammatory function [28,29].

The greatest potential relative increases in intercellular communication in obstructive HCM compared to nonobstructive HCM involved pathways between endothelial cells and endothelial cells (4 L–R pairs vs. 8 L–R pairs), endothelial cells and fibroblasts (4 L–R pairs vs. 8 L–R pairs), endothelial cells and cardiomyocytes (5 L–R pairs vs. 8 L–R pairs), and endothelial cells and neurons (7 L–R pairs vs. 10 L–R pairs; Figure 4C–E, Supplementary Figure S2). In all cases, the increase in communication pathways is due to the endothelial cells in obstructive HCM expressing additional ligands for ITGB1 (COL6A2, HSPG2, LUM). Relative to normal cells, endothelial cell communication is reduced in both types of HCM but less so in obstructive compared to nonobstructive HCM (Figure 4C–E). The induction of ECM gene expression in mature endothelial cells has been reported to be induced by hypoxic conditions [30], and thus the increase seen in nonobstructive vs. obstructive HCM may reflect relative tissue hypoxia in nonobstructive HCM.

### 2.4. Gene Ontology Enrichment Analysis of Alterations in Ligand–Receptor Pair Gene Expression Reveal Common and Distinct Alterations in Molecular Function in Nonobstructive and Obstructive HCM That Vary by Cell Type

**Figure 5:**
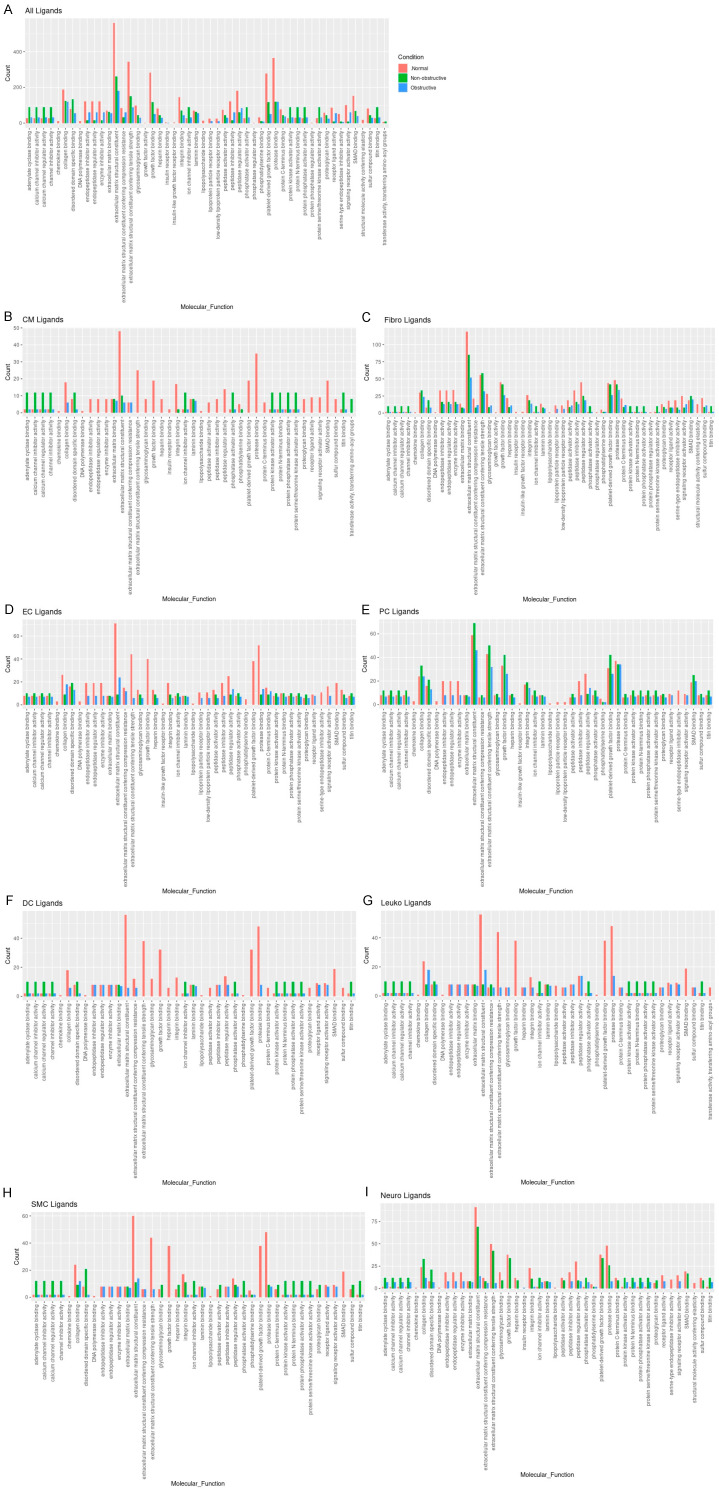
Bar plot representing the total count of ligands (in expressed ligand–receptor pairs) associated with different cellular processes in normal, nonobstructive HCM, and obstructive HCM IVS Cells. Bar color distinguishes ligand count in normal, nonobstructive HCM, or obstructive HCM conditions. (**A**) Comparison of molecular functions across all cell types. (**B**) Comparison in cardiomyocytes. (**C**) Fibroblasts. (**D**) Endothelial cells. (**E**) Pericytes. (**F**) Dendritic cells. (**G**) Leukocytes. (**H**) Smooth muscle cells. (**I**) Neurons.

To determine the molecular functions potentially affected by alterations in L–R pair gene expression in both nonobstructive and obstructive HCM across all cell types, we performed GO enrichment analysis as previously described [13,14]. We first examined ligand expression. In general, there were numerous decreases in ligands associated with many molecular functions in both types of HCM relative to normal tissue, with some variation in their degree (Figure 5A), as well as increases in various ligands associated with specific molecular functions that were unique to nonobstructive HCM. Reductions in endopeptidase inhibitor activity, endopeptidase regulator activity, enzyme inhibitor activity, extracellular matrix structural constituent, extracellular matrix structural constituent conferring tensile strength, growth factor binding, integrin binding, peptidase inhibitor activity, peptidase regulator activity, platelet-derived growth factor binding, protease binding, and SMAD binding, for example, appear to be processes affected in both types of HCM. In contrast, increases in adenylate cyclase binding, calcium channel inhibitor activity, calcium channel regulator activity, channel inhibitor activity, disordered domain specific binding, protein kinase activity, protein N-terminus binding, protein phosphatase activator activity, and titin binding are uniquely implicated in nonobstructive HCM. The analysis of cell-type-specific changes in molecular function indicate that these changes are most notable in cardiomyocytes (Figure 5B), dendritic cells (Figure 5F), leukocytes (Figure 5G), and smooth muscle cells (Figure 5H).

**Figure 6:**
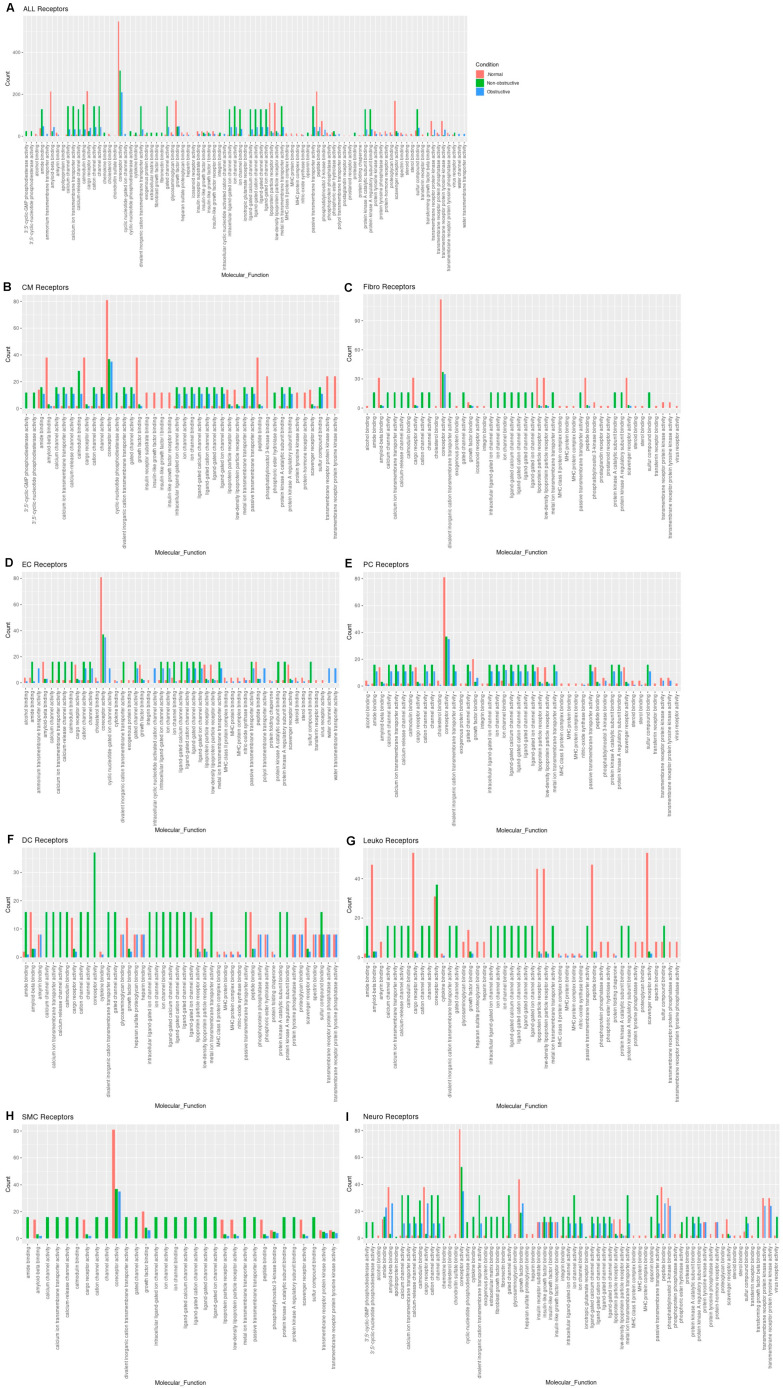
Bar plot representing the total count of receptors (in expressed ligand–receptor pairs) associated with different cellular processes in normal, nonobstructive HCM, and obstructive HCM IVS cells. Bar color distinguishes receptor count in normal or nonobstructive HCM conditions. (**A**) Comparison of molecular functions across all cell types. (**B**) Comparison in cardiomyocytes. (**C**) Fibroblasts. (**D**) Endothelial cells. (**E**) Pericytes. (**F**) Dendritic cells. (**G**) Leukocytes. (**H**) Smooth muscle cells. (**I**) Neurons.

Next, we examined the molecular functions potentially affected by alterations in receptor gene expression in both nonobstructive and obstructive HCM across all cell types by performing a GO enrichment analysis, as described previously [13,14] and above. Reductions in amyloid beta binding, cargo receptor activity, growth factor binding, lipoprotein particle receptor activity, low-density lipoprotein particle receptor activity, peptide binding, scavenger receptor activity, transmembrane receptor protein kinase activity, and transmembrane receptor protein tyrosine kinase activity are common to both nonobstructive and obstructive HCM (Figure 6A). Increases in amide binding, calcium channel activity, calcium ion transmembrane transporter activity, calcium release channel activity, calmodulin binding, cation channel activity, channel activity, divalent inorganic cation transmembrane transporter activity, gated channel activity, intracellular ligand-gated ion channel activity, ion channel activity, ion channel binding, ligand-gated calcium channel activity, ligand-gated cation channel activity, ligand-gated channel activity, ligand-gated ion channel activity, metal ion transmembrane transporter activity, passive transmembrane transporter activity, protein kinase A catalytic subunit binding, protein kinase A regulatory subunit binding, and sulfur compound binding are uniquely associated with nonobstructive HCM.

The analysis of receptor gene expression changes by specific cell type reveals both common and cell-type-specific changes in associated molecular functions (Figure 6B–I). Cardiomyocytes demonstrated the decreases in molecular functions associated with the aggregates of all cell types for each condition, as well as additional decreases in insulin receptor substrate binding, insulin-like growth factor binding, insulin-like growth factor I binding, insulin-like growth factor receptor binding, protein tyrosine kinase activity, protein-hormone receptor activity, transmembrane receptor kinase activity, and transmembrane receptor protein tyrosine kinase activity in both nonobstructive and obstructive HCM. Cardiomyocytes also showed increases in calcium channel activity, calcium ion transporter activity, calcium-release channel activity, calmodulin binding, cation channel activity, channel activity, divalent inorganic cation transmembrane transporter activity, gated channel activity, intracellular ligand-gated ion channel activity, ion channel activity, ion channel binding, ligand-gated calcium channel activity, ligand-gated cation channel activity, ligand-gated channel activity, ligand-gated ion channel activity, metal ion transmembrane transporter activity, passive transmembrane transporter activity, protein kinase A catalytic subunit binding, protein kinase A regulatory subunit binding, and sulfur compound binding as noted in the aggregate, but these changes occurred in cardiomyocytes from both nonobstructive and obstructive HCM (Figure 6B), which differ in that the aggregate nuclei population only showed these changes in nonobstructive HCM (Figure 6A). Cardiomyocytes in nonobstructive HCM showed unique increases in 3′,5-cyclic-GMP phosphodiesterase activity, 3′,5′-cyclic nucleotide phosphodiesterase activity, cyclic nucleotide phosphodiesterase activity, and phosphoric ester hydrolase activity. Fibroblasts (Figure 6C), dendritic cells (Figure 6F), leukocytes (Figure 6G), and smooth muscle cells (Figure 6H) demonstrate receptor gene expression patterns similar to those shown for the general aggregate population of nuclei. Endothelial cell receptor gene expression profiles generally paralleled the general population, except that for the various ion and calcium channel activities the increases occurred in both nonobstructive HCM and obstructive HCM, as was seen in cardiomyocytes, and for ammonium transmembrane transporter activity, cyclic nucleotide-gated ion channel activity, intracellular cyclic nucleotide activated cation channel activity, polyol transmembrane transporter activity, water channel activity, and water transmembrane transporter activity, which were uniquely increased in obstructive HCM (Figure 6D). Pericytes showed receptor gene expression patterns similar to those of the general population, except that the differences between nonobstructive and obstructive HCM were less pronounced (Figure 6E). Neurons also showed receptor gene expression patterns similar to those of the general population but shared some features with cardiomyocytes (increased 3′,5′ cyclic GMP and nucleotide phosphodiesterase and less pronounced differences between nonobstructive and obstructive HCM for other molecular functions (Figure 6I)). Overall, these findings indicate that there are changes in ligand and receptor gene expression that are cell-specific and either common or unique to each type of HCM that may provide unique, personalized, and cell-specific therapeutic targets for future therapy.

### 2.5. Fibroblast and Cardiomyocyte Subtypes Communicate Differently in Nonobstructive and Obstructive HCM

**Figure 7:**
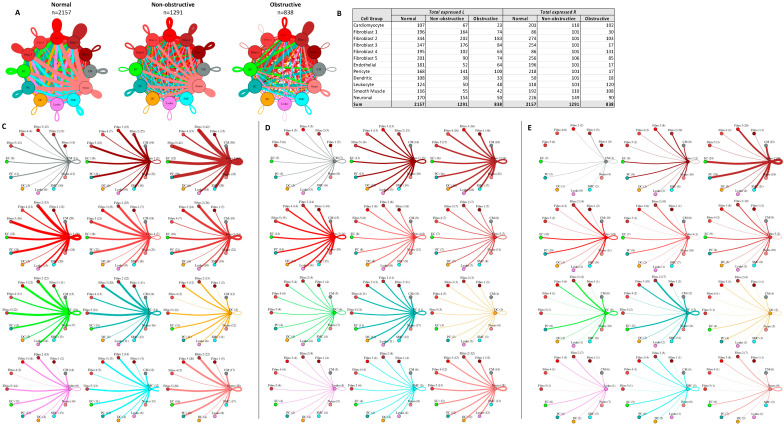
Cell–cell communication networks between fibroblast subtypes and other heart cell types in normal control, nonobstructive HCM, and obstructive HCM conditions. (**A**) Comparison of normal (left), nonobstructive (middle), and obstructive HCM (right) communication networks. Line color indicates ligand broadcast by the cell population with the same color. Lines connect to cell types that expressed cognate receptors. Line thickness is proportional to the number of uniquely expressed ligand–receptor pairs. Loops indicate communication within a cell type. (**B**) Quantity of ligands and receptors in expressed ligand–receptor pairs described by cell type and condition (normal, nonobstructive HCM, or obstructive HCM). (**C**–**E**) Cell–cell communication networks broken down by cell type and fibroblast cluster in normal control (**C**), nonobstructive (**D**), and obstructive (**E**) conditions. Figure formatting follows panel A. Numbers indicate the quantity of uniquely expressed ligand–receptor pairs between the broadcasting cell type (expressing ligand) and receiving cell type (expressing receptor).

In prior studies, fibroblast subtypes in both obstructive and nonobstructive HCM developed distinctive interaction profiles with other cells [13,14]. To compare the fibroblast interactome in both nonobstructive and obstructive HCM, we analyzed the L–R pair gene expression for all fibroblast subtypes (composed of five clusters) in conjunction with all other cell types (Figure 7). As expected, the fibroblast subtype intercellular communication networks for normal (2157), nonobstructive HCM (1291), and obstructive HCM (838) were qualitatively similar to the general cell interaction network in that fibroblast subtypes from nonobstructive HCM demonstrated reduced interactions compared to normal tissue and interactions were reduced to a greater extent in obstructive HCM tissue (Figure 7A). The greatest reduction in L–R pairs was found in ligands broadcasted from fibroblast clusters 2 and 3 to receptors on fibroblast clusters 1, 4, and 5 and leukocytes in obstructive HCM compared to nonobstructive HCM (Figure 7C,D). This reduction was due to the loss of ITGB1 receptor expression in the recipient clusters in obstructive HCM (Supplementary Figure S3). There are no cases of increased L–R interaction in obstructive HCM compared to nonobstructive HCM.

**Figure 8:**
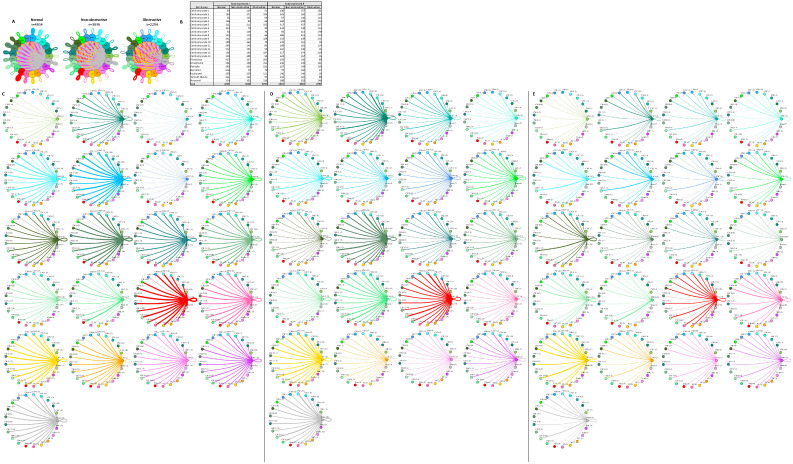
Cell–cell communication networks between cardiomyocyte subtypes and other heart cells in normal control, nonobstructive HCM, and obstructive HCM conditions. (**A**) Comparison of normal (left), nonobstructive HCM (middle), and obstructive HCM (right) communication networks. Line color indicates ligand broadcast by the cell population with the same color. Lines connect to cell types that expressed cognate receptors. Line thickness is proportional to the number of uniquely expressed ligand–receptor pairs. Loops indicate communication within a cell type. (**B**) Quantity of ligands and receptors in expressed ligand–receptor pairs described by cell type and condition (normal, nonobstructive HCM, or obstructive HCM). (**C**–**E**) Cell–cell communication networks broken down by cell type and cardiomyocyte cluster in normal control (**C**), nonobstructive HCM (**D**), and obstructive HCM (**E**) conditions. Figure formatting follows panel A. Numbers indicate the quantity of uniquely expressed ligand–receptor pairs between the broadcasting cell type (expressing ligand) and receiving cell type (expressing receptor).

Cardiomyocyte subtypes also demonstrate diversity in their intercellular interactions in normal and HCM tissue, as previously reported [13,14]. To understand how these interactions compare and contrast in nonobstructive and obstructive HCM, we analyzed the L–R pair gene expression for all cardiomyocyte subtypes in conjunction with all other cell types (Figure 8). As with the general population and the fibroblast subtype interaction networks, in the cardiomyocyte subtype interactome there was a stepwise decrease in L–R pairs from normal (n = 4854) to nonobstructive (n = 3836) to obstructive (n = 2294) HCM. Cardiomyocyte clusters 1, 2, 3, 7, and 8 did not follow this trend, however, expressing higher numbers of ligands in nonobstructive HCM compared to both normal and obstructive HCM tissue. Cardiomyocyte clusters 3 and 7 also demonstrated higher than normal numbers of ligands in obstructive HCM, but to a far lesser extent (Figure 8B–E). Cardiomyocyte clusters 3, 5, and 7 and dendritic cells expressed higher numbers of receptors than normal tissue in nonobstructive HCM. Cardiomyocyte clusters 3 and 7 also showed higher numbers of receptors in obstructive HCM compared to normal tissue, but less than those in nonobstructive HCM (Figure 8B–E). Comparing L–R pairs more directly in HCM samples indicates that cardiomyocyte cluster 10 demonstrates the greatest reduction in L–R pairs in obstructive HCM compared to nonobstructive HCM, while cardiomyocyte cluster 9 shows the greatest increase (Figure 8D,E; Supplementary Figures S4 and S5). The reduction in cardiomyocyte cluster 10 communication to dendritic cells; leukocytes; and cardiomyocyte clusters 4, 5, 8, 10, 12, 13, and 14 in obstructive HCM is driven by a loss of ITGB1 receptor expression in conjunction with a reduction in the expression of ITGB1 ligands (Supplementary Figure S4). The increase in cardiomyocyte cluster 9 communication involves cardiomyocyte clusters 7, 9, and 13 and is driven by the increased expression of the ITGB1 ligands COL6A2, LUM, and VEGFA and the ligand CALM2 (Supplementary Figure S5).

**Figure 9:**
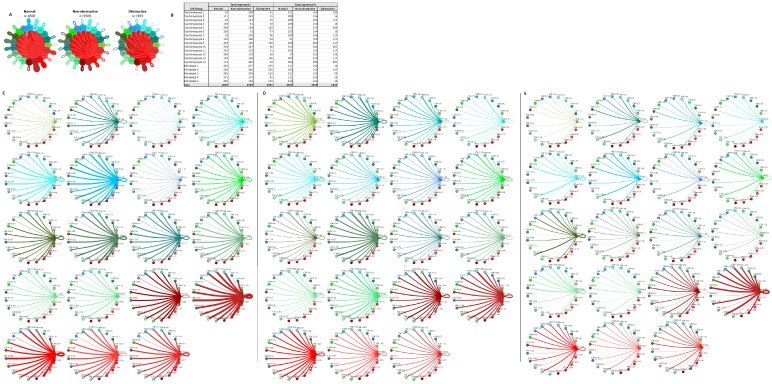
Cell–cell communication networks between cardiac fibroblast and cardiomyocyte subtypes in normal control, nonobstructive HCM, and obstructive HCM conditions. (**A**) Overall communication networks between normal, nonobstructive, and obstructive HCM cardiomyocytes and fibroblasts. Line color indicates ligand broadcast by the cell population with the same color. Lines connect to cell types which expressed cognate receptors. Line thickness is proportional to the number of uniquely expressed ligand–receptor pairs. Loops indicate communication within a cell type. (**B**) Quantity of ligands and receptors in expressed ligand–receptor pairs described by cell type and condition (normal, nonobstructive HCM, or obstructive HCM). (**C–E**) Cell–cell communication networks broken down by cardiomyocyte cluster and fibroblast cluster in normal control (**C**), nonobstructive HCM (**D**), and obstructive HCM (**E**) conditions. Figure formatting follows panel A. Numbers indicate the quantity of uniquely expressed ligand–receptor pairs between the broadcasting cell type (expressing ligand) and receiving cell type (expressing receptor).

The cardiomyocyte–fibroblast interaction network has also been postulated to play an important role in the pathogenesis of HCM through direct cellular interactions and indirect interactions with the extracellular matrix, and prior studies have shown HCM-associated alterations in cardiomyocyte–fibroblast interactions [5,13,14]. The analysis of L–R pairs among the cardiomyocytes and fibroblasts reveals the same trend seen in the general population of cells, with the greatest number of potential interactions occurring in normal cells (n = 4500) followed by nonobstructive HCM cells (n = 3509) then obstructive HCM cells (n = 1933; Figure 9A,B). Cardiomyocyte clusters 1, 2, 3, 7, and 14 do not follow this trend by expressing higher numbers of ligands in nonobstructive HCM compared to both normal and obstructive tissue. Cardiomyocyte clusters 3 and 7 also express higher numbers of ligand in obstructive HCM than normal tissue, although the numbers are less than those seen in nonobstructive tissue (Figure 9B). Cardiomyocyte clusters 8, 10, and 12 and fibroblast clusters 1 and 4 express higher levels of receptors than normal cells in nonobstructive HCM. Cardiomyocyte clusters 8 and 12 also express higher levels of receptors than normal cells, but to a lesser degree than nonobstructive HCM cells (Figure 9B–E). A more direct comparison of nonobstructive HCM and HCM reveals that the greatest difference in L–R pair number occurs in cardiomyocyte cluster 10, with reduced communication with cardiomyocyte clusters 5, 8, 10, 12, 13, and 14 and with fibroblast clusters 1, 4, and 5, largely due to the loss of the ITGB1 receptor and many ITGB1 ligands (Figure 9D,E; Supplementary Figure S6). The greatest increase in communication in the cardiomyocyte–fibroblast interaction network occurs between fibroblast cluster 2 and itself and cardiomyocyte cluster 9 and itself (Supplementary Figure S7). The increase in communication in fibroblast cluster 2 is driven by the increased expression of ITGB1 ligands such as COL1A1, FBLN1, FBN1, HPG2, LAMC1, LGALS38P, and the LRP1 receptor. The increase in communication in cardiomyocyte cluster 9 is driven by the expression of the CALM2 ligand; the ITGB1 receptor ligands COL6A2, LUM, and VEGF-A; the receptor INSR; and at least one component of the L–R pair TIMP1 and CD63 (Supplementary Figure S7).

## 3. Discussion

Previous studies have examined the transcriptional diversity and cellular composition of the normal human adult IVS [12] and the cell-specific perturbations in human HCM with LVOT obstruction [13] and in human HCM without LVOT but with end-stage heart failure prior to heart transplantation [14]. These studies have consistently shown significant transcriptional diversity associated with multiple cardiomyocyte and fibroblast subtypes and have implicated novel proteins, signaling pathways, and intercellular interactions in the pathogenesis of HCM, particularly the interaction between ITGB1 receptor expression in multiple cell types and its several extracellular-matrix-associated cognate ligands. Increased interaction with immune cells is also a notable feature in both obstructive and nonobstructive HCM [13,14]. A direct comparison of nonobstructive and obstructive HCM to identify overlapping and distinct transcriptional profiles at the single-cell level has not yet been performed, to the best of our knowledge. Our study is thus the first to directly compare samples from patients with obstructive and nonobstructive HCM and delineate overlapping and divergent patterns of gene expression at the single-cell level. In both types of HCM, there is a reduction in the intercellular interaction networks between cells, and many of the disrupted interactions involve the ITGB1 receptor and its several ECM-associated cognate ligands. The disruption of ITGB1 function in the murine myocardium is known to disrupt myocardial function [31] and alterations in the ECM that affect contractile function have been noted in HCM [32]. Alterations in ITGB1 signaling may affect biomechanical stress signals (reviewed in [33]). The reduction in the interactome is greater in obstructive HCM, however, and there are many divergently expressed genes and shifts in L–R pair gene expression patterns that likely reflect unique pathogenic mechanisms in each HCM subtype.

The GO analysis of overall changes in gene expression that diverge in nonobstructive and obstructive HCM suggest relative increases in tubulin and actin binding and muscle contraction and involve the sarcomere; myofibril; contractile fibers; and relative decreases in peptide and amide binding, aging, cell motility, and the extracellular matrix in nonobstructive HCM. The directionality of these changes may be less important than the GO categories themselves, as the change in ECM gene expression has been noted to be paradoxical—i.e., ECM gene expression has been noted to be lower in conditions where fibrosis is increased, suggesting a negative feedback loop [13,14]. The divergence in muscle contraction and sarcomere components likely reflects divergent pathogenic pathways in the IVS, with changes in muscle contraction and sarcomere elements possibly reflecting the asymmetric hypertrophy of the IVS associated with LVOT obstruction and the more severe heart failure seen in nonobstructive HCM patients undergoing cardiac transplantation. ECM changes likely reflect a difference in fibrosis in the two conditions, with greater fibrosis generally expected in end-stage, nonobstructive HCM hearts prior to transplantation.

Comparative, combined L–R pair gene expression and GO analysis allowed the direct comparison of molecular processes affected commonly and divergently in nonobstructive HCM and obstructive HCM. The common molecular functions described above likely reflect altered extracellular matrix composition, turnover, and remodeling through the action of matrix proteases, in conjunction with alterations in mechanical and growth factor-mediated signal transduction through interactions with integrin-β1, Platelet-derived growth factor (PDGF), and SMADs. PDGF is a well-known stimulator of smooth muscle proliferation [34] and thus may provide a mechanistic link to the medial vascular hypertrophy seen in HCM myocardium. SMADs are well-known mediators of fibrosis induced by TGF-β [35]. The exact mechanisms by which these alterations in molecular function directly promote the features of HCM require future study, but open further avenues for exploration and therapeutic targeting.

A striking feature of nonobstructive HCM in our study is the relative increase in a variety of signaling pathway functions over both normal and obstructive HCM conditions. Increases in adenylate cyclase binding, calcium channel inhibition, and protein kinase activation may reflect the worsened heart failure in this nonobstructive patient population [36]. Another striking feature is the skewed distribution of the altered signaling response, which is most pronounced in fibroblasts, dendritic cells, leukocytes, and smooth muscle cells, suggesting that they play a larger role in nonobstructive HCM than in obstructive HCM. These findings raise the interesting possibility that fibroblasts, dendritic cells, leukocytes and smooth muscle cells play key roles in the pathogenesis of nonobstructive HCM, perhaps through the regulation of the extracellular matrix, immune system activation and microvascular occlusion, all of which are known to occur in pathological hypertrophy and HCM [1,26,37].

The analysis of the divergence in the fibroblast interactome in obstructive HCM is likely due to a precipitous drop in ITGB1 receptor expression in several fibroblast subtypes and leukocytes. This is consistent with a reduced role for leukocytes in obstructive HCM compared to nonobstructive HCM. The analysis of the divergence in the cardiomyocyte interactome in obstructive HCM also shows the effects of the loss of ITGB1 in a variety of cardiomyocyte subtypes, leukocytes, and dendritic cells, consistent with a reduced role for leukocytes and dendritic cells in obstructive HCM compared to nonobstructive HCM. The analysis of the cardiomyocyte–fibroblast interactome also shows significant shifts in specific cardiomyocyte and fibroblast subtype due to changes in ITGB1- and LRP1-mediated interactions. A limitation of this analysis is that the identification of changes in cardiomyocyte and fibroblast subtype gene expression and interaction does not provide any spatial information. Future work linking these cardiomyocyte and fibroblast subtypes to specific histopathological locations in HCM tissue such as areas of fibrosis or myocyte disarray through spatial transcriptomics and the deconvolution of our snRNA-seq datasets with advanced bioinformatic tools [38] will facilitate the identification of appropriate target pathways for therapeutic intervention. A deeper analysis of the various perturbations in cellular interactions in HCM awaits future functional and mechanistic studies.

## 4. Materials and Methods

### 4.1. Single-Nuclei RNA-Seq Datasets

Nine of the ten patients with obstructive HCM and the associated snRNA-seq dataset have been previously described [13]. The six normal IVS organ donors and the associated snRNA-seq dataset have also been previously described [12,13,14]. The six nonobstructive HCM patients and the associated snRNA-seq dataset have also been previously described [14]. All snRNA-seq datasets are available in the Gene Expression Omnibus database under accession numbers GSE161921, GSE174691, and GSE181764.

### 4.2. Clustering of Cells by Gene Expression Pattern and Assignment of Cell Type Identity—Expand Title Here, or Break into Multiple Sections

Sequencing reads were processed using Cell Ranger version 6.0.1 [39]. Datasets were individually normalized and integrated using Seurat’s SCTransform development workflow to reduce batch effects [15]. Optimal clustering resolution was determined using Clustree version 0.4.3 [40] to identify the resolution where the number of clusters stays stable and was determined to be 0.9 for the integrated dataset. The assignment of cell identity to each cluster was performed using four separate analyses described in the following sentences. The expression of known cell-specific gene markers was used to identify major cell types, as has been done previously [10,12,13,14,25]. The top 20–30 differentially expressed genes in each cluster were also compared with cell-type gene expression markers from the PanglaoDB database https://panglaodb.se (accessed on 28 December 2021) [16] to independently assign cell types. Entire sets of differentially expressed genes for each cluster were also subjected to Ingenuity Pathway Analysis [18], and their inferred functions were used to identify cluster cell types independently. Upregulated genes from each cluster were also subject to gene ontology biological process association using GoStats [17], and these associations were used to further refine cell type assignment.

### 4.3. Trajectory Analysis and Identification of Differentially Expressed Genes

Trajectory analysis was performed using Monocle3 [19] to determine the relationship between subtypes of each cell type identified in our clustering analysis, as has been previously described [13,14]. We determined the root nodes for each cell type by hierarchical clustering prior to generating trajectories and assigning pseudotime to each nucleus. The analysis of obstructive HCM and normal nuclei together and nonobstructive HCM and normal nuclei together has been reported previously [13,14]. Each cell type was analyzed across all three conditions: normal, nonobstructive HCM, and obstructive HCM. Differentially expressed genes over trajectory paths in UMAP space (i.e., spatial autocorrelation) were determined with Monocle3 using Moran’s I statistic. Moran’s I statistic is a value that varies from −1 to 1, where −1 indicates perfect dispersion, 0 indicates no spatial autocorrelation, and 1 indicates perfect positive autocorrelation (i.e., nearby cells in have similar gene expression values in focal region of UMAP space). For each normal, nonobstructive HCM, and obstructive HCM cell type, a gene was determined to be differentially expressed over space if the associated Moran’s I statistic value was positive, paired with a significant adjusted *p*-value ≤ 0.05, and expressed in ≥1% of associated cells. Since many genes showed differential expression over space, the analysis was limited to nonobstructive HCM and obstructive HCM and further conservative filtering was performed, in which genes with Moran’s I statistic available in a single class (i.e., normal or HCM) were filtered by Moran’s I statistic values >0.1. For genes with Moran’s I statistics available in both classes (i.e., nonobstructive and obstructive HCM), genes were filtered by an absolute difference >0.1. The GO analysis of molecular function and biological process associated with differentially expressed genes was carried out using the online tools at uniprot.org/uniprotkb [41].

### 4.4. Analysis of Ligand–Receptor Pair Gene Expression to Discover Intercellular Communication Pathways

To quantify potential cardiac cell–cell communication in normal, nonobstructive HCM, and obstructive HCM hearts, cell communication networks were plotted in igraph version 1.2.6 [42] and compared on the basis of ligand–receptor pair gene expression, as we have used previously [13,14]. Our cell–cell communication networks were derived as described previously [22] using a list of 2557 human ligand–receptor pairs [23] combined with another list of 3398 human ligand–receptor pairs [24], to give a total of 3627 unique human ligand–receptor pairs, largely as described previously [13,14]. We initially analyzed the potential signaling interactions between the 8 cell types identified in our snRNA-seq data. Lines in our cell networks connect two cell types and represent expressed human ligand–receptor pairs (i.e., potential cell–cell communication between a broadcasting (ligand) and recipient (receptor) cell types. Line color in our networks represents the broadcasting ligand source. Line thickness is proportional to the number of uniquely expressed ligand–receptor pairs. Cell–cell communication networks were also analyzed by fibroblast cluster along with other cell types, by cardiomyocyte cluster and other cell types, and by fibroblast clusters and cardiomyocyte clusters. The GO analysis of differentially expressed ligand receptor pairs was performed using the R package clusterProfiler [43].

## 5. Conclusions

Human HCM, generally considered a disease of the sarcomere but known to have mitral valve abnormalities, fibrosis, and vascular abnormalities that cannot be directly explained by cardiomyocyte dysfunction, has long been postulated to involve uncharacterized interactions between cardiomyocytes and other cell types to explain these noncardiomyocyte abnormalities. A systematic comparison of these interactions in obstructive and nonobstructive HCM has not been reported until now. Here, we delineate the common mechanisms involving changes in integrin-β1 expression leading to reduced interactions between various cells and their extracellular matrix ligands. We note that this happens to a greater degree in obstructive HCM compared to nonobstructive HCM and results in differential interactions with dendritic cells and lymphocytes, implying both a difference in extracellular matrix interaction and a difference in immune system activation. Nonobstructive HCM cells also show the differential regulation of signaling pathways involving adenylate cyclase, calcium channels, and SMADs across a variety of cell types but especially in dendritic cells, lymphocytes, and smooth muscle cells, providing additional potential mechanisms by which HCM involves noncardiomyocyte cells and additional targets for therapeutic intervention.

## Figures and Tables

**Table 1:** Differentially expressed genes of visual interest in nonobstructive and obstructive HCM.

	Gene of Interest	Affected Cell Group	Expression in Non-Obstructive HCM (Compared to Obstructive)	Established HCM Gene
1	AC010680.5	Cardiomyocyte, Neuronal	Increased	
2	ALDOA	Cardiomyocyte	Increased	
3	APOD	Leukocyte	Decreased	
4	APOE	Leukocyte	Decreased	
5	ATP5B	Cardiomyocyte	Decreased	
6	ATP5E	Cardiomyocyte	Decreased	
7	ATP5G3	Cardiomyocyte	Decreased	
8	ATP5I	Cardiomyocyte	Decreased	
9	ATP5J	Cardiomyocyte	Decreased	
10	ATP5J2	Cardiomyocyte	Decreased	
11	ATP5L	Cardiomyocyte	Decreased	
12	ATP5O	Cardiomyocyte	Decreased	
13	ATPIF1	Cardiomyocyte	Decreased	
14	B2M	Cardiomyocyte	Decreased	
15	C14orf2	Cardiomyocyte	Decreased	
16	CMYA5	Cardiomyocyte	Increased	
17	COX7A1	Cardiomyocyte	Increased	
18	COX7C	Cardiomyocyte	Increased	
19	CRYAB	Cardiomyocyte	Increased	
20	CSRP3	Cardiomyocyte	Increased	Yes
21	DES	Cardiomyocyte	Increased	
22	HLA-B	Endothelial	Decreased	
23	HOOK2	Cardiomyocyte	Decreased	
24	IGFBP7	Pericyte	Decreased	
25	ITM2B	Fibroblast	Decreased	
26	KIF1C	Cardiomyocyte	Increased	
27	LUM	Fibroblast, Leukocyte	Decreased	
28	MFAP4	Fibroblast	Decreased	
29	MYH7B	Pericyte	Decreased	
30	NEAT1	Pericyte	Increased	
31	PALLD	Neuronal	Increased	
32	RGS5	Endothelial	Increased	
33	SERPINF1	Fibroblast	Decreased	
34	SLMAP	Cardiomyocyte, Pericyte	Increased	
35	TIMP1	Fibroblast, Leukocyte	Decreased	
36	USMG5	Cardiomyocyte	Decreased	
37	ZNF106	Cardiomyocyte	Increased	

## Data Availability

The datasets used in study have been previously described [12,13,14] and are available online via the Gene Expression Omnibus database under accession numbers GSE161921, GSE174691, and GSE181764.

## Supplementary Materials

Supplementary materials can be found at https://www.mdpi.com/article/10.3390/ijms23020946/s1.

## Abbreviations

HCM	Hypertrophic cardiomypathy
LVOT	Left ventricular outflow tract obstruction
snRNA-seq	Single-nucleus RNA-sequencing
IVS	Interventricular septum
ITGB1	Integrin-β1
ECM	Extracellular matrix
GO	Gene ontology
GEO	Gene Expression Omnibus
UMAP	Uniform manifold approximation and projection
L–R	Ligand–receptor
PDGF	Platelet-derived growth factor
