# Supplementary material for: Common and Distinctive Intercellular Communication Patterns in Human Obstructive and Nonobstructive Hypertrophic Cardiomyopathy
Source: Int J Mol Sci. 2022 Jan 15;23(2):946. doi: 10.3390/ijms23020946 (PMC8780670; doi:10.3390/ijms23020946)
Supplement: Supplementary file 1 [file ijms-23-00946-s001.zip › ijms-1553718-supplementary.pdf]

Supplemental Table S1. Differentially Expressed Genes in Nonobstructive and Obstructive HCM Across Cell Types

	Filtered Differentially Expressed Genes Over Space	Cell Type
1	ABCA10	Leukocyte
2	ABCA6	Leukocyte, Neuronal
3	ABCA8	Leukocyte, Neuronal
4	AC010680.5	Cardiomyocyte, Neuronal
5	ACTA1	Fibroblast, Dendritic, Leukocyte
6	ADH1B	Fibroblast, Leukocyte
7	AEBP1	Leukocyte
8	AKAP13	Neuronal
9	ALDOA	Cardiomyocyte
10	APOD	Leukocyte
11	APOE	Leukocyte
12	AQP1	Endothelial
13	ATP5B	Cardiomyocyte
14	ATP5E	Cardiomyocyte
15	ATP5G3	Cardiomyocyte
16	ATP5I	Cardiomyocyte
17	ATP5J	Cardiomyocyte
18	ATP5J2	Cardiomyocyte
19	ATP5L	Cardiomyocyte
20	ATP5O	Cardiomyocyte
21	ATPIF1	Cardiomyocyte
22	B2M	Cardiomyocyte, Endothelial, Neuronal
23	C14orf2	Cardiomyocyte
24	C1orf56	Neuronal
25	C1R	Leukocyte
26	C1S	Leukocyte
27	CCDC80	Leukocyte
28	CD55	Fibroblast, Leukocyte
29	CD74	Dendritic
30	CDC42SE1	Neuronal
31	CFD	Leukocyte
32	CLU	Leukocyte
33	CMYA5	Cardiomyocyte
34	COL1A1	Leukocyte
35	COL1A2	Leukocyte
36	COL3A1	Leukocyte
37	COX6A2	Neuronal
38	COX6C	Cardiomyocyte, Leukocyte
39	COX7A1	Cardiomyocyte
40	COX7C	Cardiomyocyte, Neuronal
41	CRYAB	Cardiomyocyte, Neuronal
42	CSRP3	Cardiomyocyte, Leukocyte, Neuronal
43	DCN	Fibroblast, Leukocyte, Neuronal
44	DES	Cardiomyocyte
45	EMC10	Cardiomyocyte
46	FABP4	Endothelial
47	FAM174A	Neuronal
48	FBLN1	Leukocyte
49	FBLN2	Leukocyte
50	FBN1	Leukocyte
51	FN1	Fibroblast, Leukocyte
52	GSN	Fibroblast, Leukocyte, Neuronal
53	HES1	Fibroblast, Leukocyte
54	HLA-B	Endothelial
55	HLA-DPA1	Dendritic
56	HLA-DPB1	Dendritic
57	HLA-DQA1	Dendritic
58	HLA-DQB1	Dendritic
59	HLA-DRA	Dendritic
60	HLA-DRB1	Dendritic
61	HLA-DRB5	Dendritic
62	HNRNPH1	Neuronal
63	HOOK2	Cardiomyocyte
64	IGFBP6	Fibroblast, Leukocyte
65	IGFBP7	Pericyte, Leukocyte
66	ITM2A	Endothelial
67	ITM2B	Fibroblast, Leukocyte
68	KIF1C	Cardiomyocyte
69	LTBP4	Leukocyte
70	LUM	Fibroblast, Leukocyte, Neuronal
71	MALAT1	Cardiomyocyte, Fibroblast, Dendritic, Neuronal
72	MEG3	Leukocyte, Smooth Muscle, Neuronal
73	MFAP4	Fibroblast, Leukocyte
74	MFAP5	Leukocyte
75	MMP2	Leukocyte
76	MS4A6A	Leukocyte
77	MYH11	Smooth Muscle
78	MYH7B	Cardiomyocyte, Pericyte, Neuronal
79	MYL12A	Cardiomyocyte, Neuronal
80	MYL2	Cardiomyocyte, Fibroblast, Endothelial, Pericyte, Dendritic, Leukocyte
81	MYL3	Neuronal
82	NDUFA1	Cardiomyocyte
83	NDUFA4	Cardiomyocyte, Fibroblast, Leukocyte, Neuronal
84	NEAT1	Cardiomyocyte, Pericyte, Leukocyte, Neuronal
85	NPPB	Cardiomyocyte
86	PALLD	Neuronal
87	PCOLCE2	Fibroblast, Leukocyte
88	PLAGL1	Neuronal
89	PLEKHH2	Neuronal
90	PRELP	Leukocyte
91	PRG4	Fibroblast, Leukocyte
92	RGS5	Endothelial, Pericyte, Neuronal
93	SCN7A	Neuronal
94	SERPINE2	Leukocyte
95	SERPINF1	Fibroblast, Leukocyte
96	SERPING1	Leukocyte
97	SLMAP	Cardiomyocyte, Pericyte, Neuronal
98	SOD3	Leukocyte
99	SORBS2	Cardiomyocyte, Pericyte, Neuronal
100	SPARCL1	Leukocyte
101	THBS4	Leukocyte
102	TIMP1	Fibroblast, Leukocyte
103	TIMP3	Leukocyte
104	TMSB10	Endothelial
105	TMSB4X	Cardiomyocyte, Neuronal
106	TNNI3	Cardiomyocyte, Fibroblast, Leukocyte
107	TNXB	Leukocyte
108	TPM1	Dendritic
109	TTN	Neuronal
110	UQCRB	Cardiomyocyte
111	UQCRQ	Cardiomyocyte
112	USMG5	Cardiomyocyte
113	VCAN	Leukocyte
114	VSIG4	Dendritic
115	ZNF106	Cardiomyocyte
116	ZNF441	Neuronal

Neuro-Fibro communication

Non-obstructive				
**Pair_Name**	**Ligand**	**Receptor**	**L_cell**	**R_cell**
CALM1_RYR2	CALM1	RYR2	Neuro	Fibro
COL1A2_CD36	COL1A2	CD36	Neuro	Fibro
COL1A2_ITGB1	COL1A2	ITGB1	Neuro	Fibro
COL4A1_ITGB1	COL4A1	ITGB1	Neuro	Fibro
COL6A1_ITGB1	COL6A1	ITGB1	Neuro	Fibro
COL6A2_ITGB1	COL6A2	ITGB1	Neuro	Fibro
FN1_ITGB1	FN1	ITGB1	Neuro	Fibro
LAMA2_ITGB1	LAMA2	ITGB1	Neuro	Fibro
LGALS1_ITGB1	LGALS1	ITGB1	Neuro	Fibro
LUM_ITGB1	LUM	ITGB1	Neuro	Fibro
S100A1_RYR2	S100A1	RYR2	Neuro	Fibro
TGM2_ITGB1	TGM2	ITGB1	Neuro	Fibro

Obstructive				
**Pair_Name**	**Ligand**	**Receptor**	**L_cell**	**R_cell**
COL6A2_ITGB1	COL6A2	ITGB1	Neuro	Fibro
LGALS1_ITGB1	LGALS1	ITGB1	Neuro	Fibro
LUM_ITGB1	LUM	ITGB1	Neuro	Fibro
TIMP1_CD63	TIMP1	CD63	Neuro	Fibro

Fibro-Leuko communication

Non-obstructive				
**Pair_Name**	**Ligand**	**Receptor**	**L_cell**	**R_cell**
CALM1_RYR2	CALM1	RYR2	Fibro	Leuko
COL1A2_CD36	COL1A2	CD36	Fibro	Leuko
COL1A2_ITGB1	COL1A2	ITGB1	Fibro	Leuko
COL3A1_ITGB1	COL3A1	ITGB1	Fibro	Leuko
COL4A1_ITGB1	COL4A1	ITGB1	Fibro	Leuko
COL6A1_ITGB1	COL6A1	ITGB1	Fibro	Leuko
COL6A2_ITGB1	COL6A2	ITGB1	Fibro	Leuko
COL6A3_ITGB1	COL6A3	ITGB1	Fibro	Leuko
FN1_ITGB1	FN1	ITGB1	Fibro	Leuko
LAMA2_ITGB1	LAMA2	ITGB1	Fibro	Leuko
LGALS1_ITGB1	LGALS1	ITGB1	Fibro	Leuko
LUM_ITGB1	LUM	ITGB1	Fibro	Leuko
S100A1_RYR2	S100A1	RYR2	Fibro	Leuko
TIMP1_CD63	TIMP1	CD63	Fibro	Leuko

Obstructive				
**Pair_Name**	**Ligand**	**Receptor**	**L_cell**	**R_cell**
APP_CD74	APP	CD74	Fibro	Leuko
COL1A2_CD36	COL1A2	CD36	Fibro	Leuko
TIMP1_CD63	TIMP1	CD63	Fibro	Leuko

Fibro-DC communication

Non-obstructive				
**Pair_Name**	**Ligand**	**Receptor**	**L_cell**	**R_cell**
CALM1_RYR2	CALM1	RYR2	Fibro	DC
COL1A2_CD36	COL1A2	CD36	Fibro	DC
COL1A2_ITGB1	COL1A2	ITGB1	Fibro	DC
COL3A1_ITGB1	COL3A1	ITGB1	Fibro	DC
COL4A1_ITGB1	COL4A1	ITGB1	Fibro	DC
COL6A1_ITGB1	COL6A1	ITGB1	Fibro	DC
COL6A2_ITGB1	COL6A2	ITGB1	Fibro	DC
COL6A3_ITGB1	COL6A3	ITGB1	Fibro	DC
FN1_ITGB1	FN1	ITGB1	Fibro	DC
LAMA2_ITGB1	LAMA2	ITGB1	Fibro	DC
LGALS1_ITGB1	LGALS1	ITGB1	Fibro	DC
LUM_ITGB1	LUM	ITGB1	Fibro	DC
S100A1_RYR2	S100A1	RYR2	Fibro	DC
TIMP1_CD63	TIMP1	CD63	Fibro	DC

Obstructive				
**Pair_Name**	**Ligand**	**Receptor**	**L_cell**	**R_cell**
APP_CD74	APP	CD74	Fibro	DC
COL1A2_CD36	COL1A2	CD36	Fibro	DC
LGALS1_PTPRC	LGALS1	PTPRC	Fibro	DC
TIMP1_CD63	TIMP1	CD63	Fibro	DC

Neuro-DC communication

Non-obstructive				
**Pair_Name**	**Ligand**	**Receptor**	**L_cell**	**R_cell**
CALM1_RYR2	CALM1	RYR2	Neuro	DC
COL1A2_CD36	COL1A2	CD36	Neuro	DC
COL1A2_ITGB1	COL1A2	ITGB1	Neuro	DC
COL4A1_ITGB1	COL4A1	ITGB1	Neuro	DC
COL6A1_ITGB1	COL6A1	ITGB1	Neuro	DC
COL6A2_ITGB1	COL6A2	ITGB1	Neuro	DC
FN1_ITGB1	FN1	ITGB1	Neuro	DC
LAMA2_ITGB1	LAMA2	ITGB1	Neuro	DC
LGALS1_ITGB1	LGALS1	ITGB1	Neuro	DC
LUM_ITGB1	LUM	ITGB1	Neuro	DC
S100A1_RYR2	S100A1	RYR2	Neuro	DC
TGM2_ITGB1	TGM2	ITGB1	Neuro	DC

Obstructive				
**Pair_Name**	**Ligand**	**Receptor**	**L_cell**	**R_cell**
LGALS1_PTPRC	LGALS1	PTPRC	Neuro	DC
TIMP1_CD63	TIMP1	CD63	Neuro	DC

Supplemental Figure S1. Reduced Ligand-Receptor Pair Gene Expression In Obstructive vs. Nonobstructive HCM

EC-EC communication

Non-obstructive				
**Pair_Name**	**Ligand**	**Receptor**	**L_cell**	**R_cell**
CALM1_RYR2	CALM1	RYR2	EC	EC
FN1_ITGB1	FN1	ITGB1	EC	EC
LGALS1_ITGB1	LGALS1	ITGB1	EC	EC
S100A1_RYR2	S100A1	RYR2	EC	EC

Obstructive				
**Pair_Name**	**Ligand**	**Receptor**	**L_cell**	**R_cell**
CALM1_AQP1	CALM1	AQP1	EC	EC
CALM2_AQP1	CALM2	AQP1	EC	EC
COL6A2_ITGB1	COL6A2	ITGB1	EC	EC
FN1_ITGB1	FN1	ITGB1	EC	EC
HSPG2_ITGB1	HSPG2	ITGB1	EC	EC
LGALS1_ITGB1	LGALS1	ITGB1	EC	EC
LUM_ITGB1	LUM	ITGB1	EC	EC
TIMP1_CD63	TIMP1	CD63	EC	EC

EC-PC communication

Non-obstructive				
**Pair_Name**	**Ligand**	**Receptor**	**L_cell**	**R_cell**
CALM1_RYR2	CALM1	RYR2	EC	PC
FN1_ITGB1	FN1	ITGB1	EC	PC
LGALS1_ITGB1	LGALS1	ITGB1	EC	PC
S100A1_RYR2	S100A1	RYR2	EC	PC

Obstructive				
**Pair_Name**	**Ligand**	**Receptor**	**L_cell**	**R_cell**
CALM1_RYR2	CALM1	RYR2	EC	PC
COL6A2_ITGB1	COL6A2	ITGB1	EC	PC
FN1_ITGB1	FN1	ITGB1	EC	PC
HSPG2_ITGB1	HSPG2	ITGB1	EC	PC
LGALS1_ITGB1	LGALS1	ITGB1	EC	PC
LUM_ITGB1	LUM	ITGB1	EC	PC
S100A1_RYR2	S100A1	RYR2	EC	PC
TIMP1_CD63	TIMP1	CD63	EC	PC

EC-CM communication

Non-obstructive				
**Pair_Name**	**Ligand**	**Receptor**	**L_cell**	**R_cell**
CALM1_PDE1C	CALM1	PDE1C	EC	CM
CALM1_RYR2	CALM1	RYR2	EC	CM
FN1_ITGB1	FN1	ITGB1	EC	CM
LGALS1_ITGB1	LGALS1	ITGB1	EC	CM
S100A1_RYR2	S100A1	RYR2	EC	CM

Obstructive				
**Pair_Name**	**Ligand**	**Receptor**	**L_cell**	**R_cell**
CALM1_RYR2	CALM1	RYR2	EC	CM
COL6A2_ITGB1	COL6A2	ITGB1	EC	CM
FN1_ITGB1	FN1	ITGB1	EC	CM
HSPG2_ITGB1	HSPG2	ITGB1	EC	CM
LGALS1_ITGB1	LGALS1	ITGB1	EC	CM
LUM_ITGB1	LUM	ITGB1	EC	CM
S100A1_RYR2	S100A1	RYR2	EC	CM
TIMP1_CD63	TIMP1	CD63	EC	CM

EC-Neuro communication

Non-obstructive				
**Pair_Name**	**Ligand**	**Receptor**	**L_cell**	**R_cell**
CALM1_PDE1C	CALM1	PDE1C	EC	Neuro
CALM1_RYR2	CALM1	RYR2	EC	Neuro
FN1_ITGAV	FN1	ITGAV	EC	Neuro
FN1_ITGB1	FN1	ITGB1	EC	Neuro
HLA-B_CANX	HLA-B	CANX	EC	Neuro
LGALS1_ITGB1	LGALS1	ITGB1	EC	Neuro
S100A1_RYR2	S100A1	RYR2	EC	Neuro

Obstructive				
**Pair_Name**	**Ligand**	**Receptor**	**L_cell**	**R_cell**
CALM1_INSR	CALM1	INSR	EC	Neuro
CALM1_RYR2	CALM1	RYR2	EC	Neuro
CALM2_INSR	CALM2	INSR	EC	Neuro
COL6A2_ITGB1	COL6A2	ITGB1	EC	Neuro
FN1_ITGB1	FN1	ITGB1	EC	Neuro
HSPG2_ITGB1	HSPG2	ITGB1	EC	Neuro
LGALS1_ITGB1	LGALS1	ITGB1	EC	Neuro
LUM_ITGB1	LUM	ITGB1	EC	Neuro
S100A1_RYR2	S100A1	RYR2	EC	Neuro
TIMP1_CD63	TIMP1	CD63	EC	Neuro

Supplemental Figure S2. Increased Ligand-Receptor Pair Gene Expression In Obstructive vs. Nonobstructive HCM

Non-obstructive				
**Pair_Name**	**Ligand**	**Receptor**	**L_cell**	**R_cell**
CALM1_RYR2	CALM1	RYR2	Fibro 2	Fibro 1
COL1A2_CD36	COL1A2	CD36	Fibro 2	Fibro 1
COL1A2_ITGB1	COL1A2	ITGB1	Fibro 2	Fibro 1
COL3A1_ITGB1	COL3A1	ITGB1	Fibro 2	Fibro 1
COL4A1_ITGB1	COL4A1	ITGB1	Fibro 2	Fibro 1
COL6A1_ITGB1	COL6A1	ITGB1	Fibro 2	Fibro 1
COL6A2_ITGB1	COL6A2	ITGB1	Fibro 2	Fibro 1
COL6A3_ITGB1	COL6A3	ITGB1	Fibro 2	Fibro 1
FN1_ITGB1	FN1	ITGB1	Fibro 2	Fibro 1
LAMA2_ITGB1	LAMA2	ITGB1	Fibro 2	Fibro 1
LGALS1_ITGB1	LGALS1	ITGB1	Fibro 2	Fibro 1
LUM_ITGB1	LUM	ITGB1	Fibro 2	Fibro 1
S100A1_RYR2	S100A1	RYR2	Fibro 2	Fibro 1
TIMP1_CD63	TIMP1	CD63	Fibro 2	Fibro 1
TIMP2_ITGB1	TIMP2	ITGB1	Fibro 2	Fibro 1
VCAN_ITGB1	VCAN	ITGB1	Fibro 2	Fibro 1
CALM1_RYR2	CALM1	RYR2	Fibro 2	Fibro 4
COL1A2_CD36	COL1A2	CD36	Fibro 2	Fibro 4
COL1A2_ITGB1	COL1A2	ITGB1	Fibro 2	Fibro 4
COL3A1_ITGB1	COL3A1	ITGB1	Fibro 2	Fibro 4
COL4A1_ITGB1	COL4A1	ITGB1	Fibro 2	Fibro 4
COL6A1_ITGB1	COL6A1	ITGB1	Fibro 2	Fibro 4
COL6A2_ITGB1	COL6A2	ITGB1	Fibro 2	Fibro 4
COL6A3_ITGB1	COL6A3	ITGB1	Fibro 2	Fibro 4
FN1_ITGB1	FN1	ITGB1	Fibro 2	Fibro 4
LAMA2_ITGB1	LAMA2	ITGB1	Fibro 2	Fibro 4
LGALS1_ITGB1	LGALS1	ITGB1	Fibro 2	Fibro 4
LUM_ITGB1	LUM	ITGB1	Fibro 2	Fibro 4
S100A1_RYR2	S100A1	RYR2	Fibro 2	Fibro 4
TIMP1_CD63	TIMP1	CD63	Fibro 2	Fibro 4
TIMP2_ITGB1	TIMP2	ITGB1	Fibro 2	Fibro 4
VCAN_ITGB1	VCAN	ITGB1	Fibro 2	Fibro 4
CALM1_RYR2	CALM1	RYR2	Fibro 2	Fibro 5
COL1A2_CD36	COL1A2	CD36	Fibro 2	Fibro 5
COL1A2_ITGB1	COL1A2	ITGB1	Fibro 2	Fibro 5
COL3A1_ITGB1	COL3A1	ITGB1	Fibro 2	Fibro 5
COL4A1_ITGB1	COL4A1	ITGB1	Fibro 2	Fibro 5
COL6A1_ITGB1	COL6A1	ITGB1	Fibro 2	Fibro 5
COL6A2_ITGB1	COL6A2	ITGB1	Fibro 2	Fibro 5
COL6A3_ITGB1	COL6A3	ITGB1	Fibro 2	Fibro 5
FN1_ITGB1	FN1	ITGB1	Fibro 2	Fibro 5
LAMA2_ITGB1	LAMA2	ITGB1	Fibro 2	Fibro 5
LAMA2_RPSA	LAMA2	RPSA	Fibro 2	Fibro 5
LGALS1_ITGB1	LGALS1	ITGB1	Fibro 2	Fibro 5
LUM_ITGB1	LUM	ITGB1	Fibro 2	Fibro 5
S100A1_RYR2	S100A1	RYR2	Fibro 2	Fibro 5
TIMP1_CD63	TIMP1	CD63	Fibro 2	Fibro 5
TIMP2_ITGB1	TIMP2	ITGB1	Fibro 2	Fibro 5
VCAN_ITGB1	VCAN	ITGB1	Fibro 2	Fibro 5
CALM1_RYR2	CALM1	RYR2	Fibro 2	Leuko
COL1A2_CD36	COL1A2	CD36	Fibro 2	Leuko
COL1A2_ITGB1	COL1A2	ITGB1	Fibro 2	Leuko
COL3A1_ITGB1	COL3A1	ITGB1	Fibro 2	Leuko
COL4A1_ITGB1	COL4A1	ITGB1	Fibro 2	Leuko
COL6A1_ITGB1	COL6A1	ITGB1	Fibro 2	Leuko
COL6A2_ITGB1	COL6A2	ITGB1	Fibro 2	Leuko
COL6A3_ITGB1	COL6A3	ITGB1	Fibro 2	Leuko
FN1_ITGB1	FN1	ITGB1	Fibro 2	Leuko
LAMA2_ITGB1	LAMA2	ITGB1	Fibro 2	Leuko
LGALS1_ITGB1	LGALS1	ITGB1	Fibro 2	Leuko
LUM_ITGB1	LUM	ITGB1	Fibro 2	Leuko
S100A1_RYR2	S100A1	RYR2	Fibro 2	Leuko
TIMP1_CD63	TIMP1	CD63	Fibro 2	Leuko
TIMP2_ITGB1	TIMP2	ITGB1	Fibro 2	Leuko
VCAN_ITGB1	VCAN	ITGB1	Fibro 2	Leuko
CALM1_RYR2	CALM1	RYR2	Fibro 3	Fibro 2
COL1A2_CD36	COL1A2	CD36	Fibro 3	Fibro 2
COL1A2_ITGB1	COL1A2	ITGB1	Fibro 3	Fibro 2
COL3A1_ITGB1	COL3A1	ITGB1	Fibro 3	Fibro 2
COL4A1_ITGB1	COL4A1	ITGB1	Fibro 3	Fibro 2
COL6A1_ITGB1	COL6A1	ITGB1	Fibro 3	Fibro 2
COL6A2_ITGB1	COL6A2	ITGB1	Fibro 3	Fibro 2
COL6A3_ITGB1	COL6A3	ITGB1	Fibro 3	Fibro 2
FN1_ITGB1	FN1	ITGB1	Fibro 3	Fibro 2
LAMA2_ITGB1	LAMA2	ITGB1	Fibro 3	Fibro 2
LGALS1_ITGB1	LGALS1	ITGB1	Fibro 3	Fibro 2
LUM_ITGB1	LUM	ITGB1	Fibro 3	Fibro 2
S100A1_RYR2	S100A1	RYR2	Fibro 3	Fibro 2
TIMP1_CD63	TIMP1	CD63	Fibro 3	Fibro 2
CALM1_RYR2	CALM1	RYR2	Fibro 3	Fibro 4
COL1A2_CD36	COL1A2	CD36	Fibro 3	Fibro 4
COL1A2_ITGB1	COL1A2	ITGB1	Fibro 3	Fibro 4
COL3A1_ITGB1	COL3A1	ITGB1	Fibro 3	Fibro 4
COL4A1_ITGB1	COL4A1	ITGB1	Fibro 3	Fibro 4
COL6A1_ITGB1	COL6A1	ITGB1	Fibro 3	Fibro 4
COL6A2_ITGB1	COL6A2	ITGB1	Fibro 3	Fibro 4
COL6A3_ITGB1	COL6A3	ITGB1	Fibro 3	Fibro 4
FN1_ITGB1	FN1	ITGB1	Fibro 3	Fibro 4
LAMA2_ITGB1	LAMA2	ITGB1	Fibro 3	Fibro 4
LGALS1_ITGB1	LGALS1	ITGB1	Fibro 3	Fibro 4
LUM_ITGB1	LUM	ITGB1	Fibro 3	Fibro 4
S100A1_RYR2	S100A1	RYR2	Fibro 3	Fibro 4
TIMP1_CD63	TIMP1	CD63	Fibro 3	Fibro 4
CALM1_RYR2	CALM1	RYR2	Fibro 3	Fibro 5
COL1A2_CD36	COL1A2	CD36	Fibro 3	Fibro 5
COL1A2_ITGB1	COL1A2	ITGB1	Fibro 3	Fibro 5
COL3A1_ITGB1	COL3A1	ITGB1	Fibro 3	Fibro 5
COL4A1_ITGB1	COL4A1	ITGB1	Fibro 3	Fibro 5
COL6A1_ITGB1	COL6A1	ITGB1	Fibro 3	Fibro 5
COL6A2_ITGB1	COL6A2	ITGB1	Fibro 3	Fibro 5
COL6A3_ITGB1	COL6A3	ITGB1	Fibro 3	Fibro 5
FN1_ITGB1	FN1	ITGB1	Fibro 3	Fibro 5
LAMA2_ITGB1	LAMA2	ITGB1	Fibro 3	Fibro 5
LAMA2_RPSA	LAMA2	RPSA	Fibro 3	Fibro 5
LGALS1_ITGB1	LGALS1	ITGB1	Fibro 3	Fibro 5
LUM_ITGB1	LUM	ITGB1	Fibro 3	Fibro 5
S100A1_RYR2	S100A1	RYR2	Fibro 3	Fibro 5
TIMP1_CD63	TIMP1	CD63	Fibro 3	Fibro 5
CALM1_RYR2	CALM1	RYR2	Fibro 3	Leuko
COL1A2_CD36	COL1A2	CD36	Fibro 3	Leuko
COL1A2_ITGB1	COL1A2	ITGB1	Fibro 3	Leuko
COL3A1_ITGB1	COL3A1	ITGB1	Fibro 3	Leuko
COL4A1_ITGB1	COL4A1	ITGB1	Fibro 3	Leuko
COL6A1_ITGB1	COL6A1	ITGB1	Fibro 3	Leuko
COL6A2_ITGB1	COL6A2	ITGB1	Fibro 3	Leuko
COL6A3_ITGB1	COL6A3	ITGB1	Fibro 3	Leuko
FN1_ITGB1	FN1	ITGB1	Fibro 3	Leuko
LAMA2_ITGB1	LAMA2	ITGB1	Fibro 3	Leuko
LGALS1_ITGB1	LGALS1	ITGB1	Fibro 3	Leuko
LUM_ITGB1	LUM	ITGB1	Fibro 3	Leuko
S100A1_RYR2	S100A1	RYR2	Fibro 3	Leuko
TIMP1_CD63	TIMP1	CD63	Fibro 3	Leuko

Obstructive				
**Pair_Name**	**Ligand**	**Receptor**	**L_cell**	**R_cell**
COL1A1_CD36	COL1A1	CD36	Fibro 2	Fibro 1
COL1A2_CD36	COL1A2	CD36	Fibro 2	Fibro 1
TIMP1_CD63	TIMP1	CD63	Fibro 2	Fibro 1
COL1A1_CD36	COL1A1	CD36	Fibro 2	Fibro 4
COL1A2_CD36	COL1A2	CD36	Fibro 2	Fibro 4
TIMP1_CD63	TIMP1	CD63	Fibro 2	Fibro 4
COL1A1_CD36	COL1A1	CD36	Fibro 2	Fibro 5
COL1A2_CD36	COL1A2	CD36	Fibro 2	Fibro 5
TIMP1_CD63	TIMP1	CD63	Fibro 2	Fibro 5
APP_CD74	APP	CD74	Fibro 2	Leuko
COL1A1_CD36	COL1A1	CD36	Fibro 2	Leuko
COL1A2_CD36	COL1A2	CD36	Fibro 2	Leuko
TIMP1_CD63	TIMP1	CD63	Fibro 2	Leuko
COL1A2_CD36	COL1A2	CD36	Fibro 3	Fibro 1
TIMP1_CD63	TIMP1	CD63	Fibro 3	Fibro 1
COL1A2_CD36	COL1A2	CD36	Fibro 3	Fibro 4
TIMP1_CD63	TIMP1	CD63	Fibro 3	Fibro 4
COL1A2_CD36	COL1A2	CD36	Fibro 3	Fibro 5
TIMP1_CD63	TIMP1	CD63	Fibro 3	Fibro 5
COL1A2_CD36	COL1A2	CD36	Fibro 3	Leuko
TIMP1_CD63	TIMP1	CD63	Fibro 3	Leuko

Supplemental Figure S3. Reduced Fibroblast Subtype Interactome Ligand-Receptor Pair Gene Expression in Obstructive vs. Nonobstructive HCM

Non-obstructive				
**Pair-Name**	**Ligand**	**Receptor**	**L_cell**	**R_cell**
CALM1_RYR2	CALM1	RYR2	CM 10	DC
COL1A2_CD36	COL1A2	CD36	CM 10	DC
COL1A2_ITGB1	COL1A2	ITGB1	CM 10	DC
COL3A1_ITGB1	COL3A1	ITGB1	CM 10	DC
COL4A1_ITGB1	COL4A1	ITGB1	CM 10	DC
COL6A1_ITGB1	COL6A1	ITGB1	CM 10	DC
COL6A2_ITGB1	COL6A2	ITGB1	CM 10	DC
COL6A3_ITGB1	COL6A3	ITGB1	CM 10	DC
FN1_ITGB1	FN1	ITGB1	CM 10	DC
LAMA2_ITGB1	LAMA2	ITGB1	CM 10	DC
LGALS1_ITGB1	LGALS1	ITGB1	CM 10	DC
LUM_ITGB1	LUM	ITGB1	CM 10	DC
MIF_CD74	MIF	CD74	CM 10	DC
S100A1_RYR2	S100A1	RYR2	CM 10	DC
TGM2_ITGB1	TGM2	ITGB1	CM 10	DC
TIMP1_CD63	TIMP1	CD63	CM 10	DC
CALM1_RYR2	CALM1	RYR2	CM 10	Leuko
COL1A2_CD36	COL1A2	CD36	CM 10	Leuko
COL1A2_ITGB1	COL1A2	ITGB1	CM 10	Leuko
COL3A1_ITGB1	COL3A1	ITGB1	CM 10	Leuko
COL4A1_ITGB1	COL4A1	ITGB1	CM 10	Leuko
COL6A1_ITGB1	COL6A1	ITGB1	CM 10	Leuko
COL6A2_ITGB1	COL6A2	ITGB1	CM 10	Leuko
COL6A3_ITGB1	COL6A3	ITGB1	CM 10	Leuko
FN1_ITGB1	FN1	ITGB1	CM 10	Leuko
LAMA2_ITGB1	LAMA2	ITGB1	CM 10	Leuko
LGALS1_ITGB1	LGALS1	ITGB1	CM 10	Leuko
LUM_ITGB1	LUM	ITGB1	CM 10	Leuko
S100A1_RYR2	S100A1	RYR2	CM 10	Leuko
TGM2_ITGB1	TGM2	ITGB1	CM 10	Leuko
TIMP1_CD63	TIMP1	CD63	CM 10	Leuko
CALM1_CACNA1C	CALM1	CACNA1C	CM 10	CM 4
CALM1_PDE1C	CALM1	PDE1C	CM 10	CM 4
CALM1_RYR2	CALM1	RYR2	CM 10	CM 4
CALM2_CACNA1C	CALM2	CACNA1C	CM 10	CM 4
CALM2_PDE1C	CALM2	PDE1C	CM 10	CM 4
COL1A2_CD36	COL1A2	CD36	CM 10	CM 4
COL1A2_ITGB1	COL1A2	ITGB1	CM 10	CM 4
COL3A1_ITGB1	COL3A1	ITGB1	CM 10	CM 4
COL4A1_ITGB1	COL4A1	ITGB1	CM 10	CM 4
COL6A1_ITGB1	COL6A1	ITGB1	CM 10	CM 4
COL6A2_ITGB1	COL6A2	ITGB1	CM 10	CM 4
COL6A3_ITGB1	COL6A3	ITGB1	CM 10	CM 4
FN1_ITGB1	FN1	ITGB1	CM 10	CM 4
LAMA2_ITGB1	LAMA2	ITGB1	CM 10	CM 4
LGALS1_ITGB1	LGALS1	ITGB1	CM 10	CM 4
LUM_ITGB1	LUM	ITGB1	CM 10	CM 4
S100A1_RYR2	S100A1	RYR2	CM 10	CM 4
TGM2_ITGB1	TGM2	ITGB1	CM 10	CM 4
CALM1_CACNA1C	CALM1	CACNA1C	CM 10	CM 5
CALM1_INSR	CALM1	INSR	CM 10	CM 5
CALM1_PDE1C	CALM1	PDE1C	CM 10	CM 5
CALM1_RYR2	CALM1	RYR2	CM 10	CM 5
CALM2_CACNA1C	CALM2	CACNA1C	CM 10	CM 5
CALM2_INSR	CALM2	INSR	CM 10	CM 5
CALM2_PDE1C	CALM2	PDE1C	CM 10	CM 5
COL1A2_CD36	COL1A2	CD36	CM 10	CM 5
COL1A2_ITGAV	COL1A2	ITGAV	CM 10	CM 5
COL1A2_ITGB1	COL1A2	ITGB1	CM 10	CM 5
COL3A1_ITGB1	COL3A1	ITGB1	CM 10	CM 5
COL4A1_ITGAV	COL4A1	ITGAV	CM 10	CM 5
COL4A1_ITGB1	COL4A1	ITGB1	CM 10	CM 5
COL6A1_ITGB1	COL6A1	ITGB1	CM 10	CM 5
COL6A2_ITGB1	COL6A2	ITGB1	CM 10	CM 5
COL6A3_ITGB1	COL6A3	ITGB1	CM 10	CM 5
FN1_ITGAV	FN1	ITGAV	CM 10	CM 5
FN1_ITGB1	FN1	ITGB1	CM 10	CM 5
LAMA2_ITGA7	LAMA2	ITGA7	CM 10	CM 5
LAMA2_ITGB1	LAMA2	ITGB1	CM 10	CM 5
LAMA2_RPSA	LAMA2	RPSA	CM 10	CM 5
LGALS1_ITGB1	LGALS1	ITGB1	CM 10	CM 5
LUM_ITGB1	LUM	ITGB1	CM 10	CM 5
MFGE8_ITGAV	MFGE8	ITGAV	CM 10	CM 5
S100A1_RYR2	S100A1	RYR2	CM 10	CM 5
SORBS1_INSR	SORBS1	INSR	CM 10	CM 5
TGM2_ITGB1	TGM2	ITGB1	CM 10	CM 5
TIMP1_CD63	TIMP1	CD63	CM 10	CM 5
CALM1_CACNA1C	CALM1	CACNA1C	CM 10	CM 8
CALM1_PDE1C	CALM1	PDE1C	CM 10	CM 8
CALM1_RYR2	CALM1	RYR2	CM 10	CM 8
CALM2_CACNA1C	CALM2	CACNA1C	CM 10	CM 8
CALM2_PDE1C	CALM2	PDE1C	CM 10	CM 8
COL1A2_CD36	COL1A2	CD36	CM 10	CM 8
COL1A2_ITGB1	COL1A2	ITGB1	CM 10	CM 8
COL3A1_ITGB1	COL3A1	ITGB1	CM 10	CM 8
COL4A1_ITGB1	COL4A1	ITGB1	CM 10	CM 8
COL6A1_ITGB1	COL6A1	ITGB1	CM 10	CM 8
COL6A2_ITGB1	COL6A2	ITGB1	CM 10	CM 8
COL6A3_ITGB1	COL6A3	ITGB1	CM 10	CM 8
FN1_ITGB1	FN1	ITGB1	CM 10	CM 8
LAMA2_ITGB1	LAMA2	ITGB1	CM 10	CM 8
LGALS1_ITGB1	LGALS1	ITGB1	CM 10	CM 8
LUM_ITGB1	LUM	ITGB1	CM 10	CM 8
S100A1_RYR2	S100A1	RYR2	CM 10	CM 8
TGM2_ITGB1	TGM2	ITGB1	CM 10	CM 8
TIMP1_CD63	TIMP1	CD63	CM 10	CM 8
CALM1_RYR2	CALM1	RYR2	CM 10	CM 10
COL1A2_CD36	COL1A2	CD36	CM 10	CM 10
COL1A2_ITGB1	COL1A2	ITGB1	CM 10	CM 10
COL3A1_ITGB1	COL3A1	ITGB1	CM 10	CM 10
COL4A1_ITGB1	COL4A1	ITGB1	CM 10	CM 10
COL6A1_ITGB1	COL6A1	ITGB1	CM 10	CM 10
COL6A2_ITGB1	COL6A2	ITGB1	CM 10	CM 10
COL6A3_ITGB1	COL6A3	ITGB1	CM 10	CM 10
FN1_ITGB1	FN1	ITGB1	CM 10	CM 10
LAMA2_ITGB1	LAMA2	ITGB1	CM 10	CM 10
LGALS1_ITGB1	LGALS1	ITGB1	CM 10	CM 10
LUM_ITGB1	LUM	ITGB1	CM 10	CM 10
S100A1_RYR2	S100A1	RYR2	CM 10	CM 10
TGM2_ITGB1	TGM2	ITGB1	CM 10	CM 10
TIMP1_CD63	TIMP1	CD63	CM 10	CM 10
CALM1_RYR2	CALM1	RYR2	CM 10	CM 12
COL1A2_CD36	COL1A2	CD36	CM 10	CM 12
COL1A2_ITGB1	COL1A2	ITGB1	CM 10	CM 12
COL3A1_ITGB1	COL3A1	ITGB1	CM 10	CM 12
COL4A1_ITGB1	COL4A1	ITGB1	CM 10	CM 12
COL6A1_ITGB1	COL6A1	ITGB1	CM 10	CM 12
COL6A2_ITGB1	COL6A2	ITGB1	CM 10	CM 12
COL6A3_ITGB1	COL6A3	ITGB1	CM 10	CM 12
FN1_ITGB1	FN1	ITGB1	CM 10	CM 12
LAMA2_ITGB1	LAMA2	ITGB1	CM 10	CM 12
LGALS1_ITGB1	LGALS1	ITGB1	CM 10	CM 12
LUM_ITGB1	LUM	ITGB1	CM 10	CM 12
S100A1_RYR2	S100A1	RYR2	CM 10	CM 12
TGM2_ITGB1	TGM2	ITGB1	CM 10	CM 12
TIMP1_CD63	TIMP1	CD63	CM 10	CM 12
CALM1_CACNA1C	CALM1	CACNA1C	CM 10	CM 13
CALM1_PDE1C	CALM1	PDE1C	CM 10	CM 13
CALM1_RYR2	CALM1	RYR2	CM 10	CM 13
CALM2_CACNA1C	CALM2	CACNA1C	CM 10	CM 13
CALM2_PDE1C	CALM2	PDE1C	CM 10	CM 13
COL1A2_CD36	COL1A2	CD36	CM 10	CM 13
COL1A2_ITGAV	COL1A2	ITGAV	CM 10	CM 13
COL1A2_ITGB1	COL1A2	ITGB1	CM 10	CM 13
COL3A1_ITGB1	COL3A1	ITGB1	CM 10	CM 13
COL4A1_ITGAV	COL4A1	ITGAV	CM 10	CM 13
COL4A1_ITGB1	COL4A1	ITGB1	CM 10	CM 13
COL6A1_ITGB1	COL6A1	ITGB1	CM 10	CM 13
COL6A2_ITGB1	COL6A2	ITGB1	CM 10	CM 13
COL6A3_ITGB1	COL6A3	ITGB1	CM 10	CM 13
FN1_ITGAV	FN1	ITGAV	CM 10	CM 13
FN1_ITGB1	FN1	ITGB1	CM 10	CM 13
LAMA2_ITGB1	LAMA2	ITGB1	CM 10	CM 13
LAMA2_RPSA	LAMA2	RPSA	CM 10	CM 13
LGALS1_ITGB1	LGALS1	ITGB1	CM 10	CM 13
LUM_ITGB1	LUM	ITGB1	CM 10	CM 13
MFGE8_ITGAV	MFGE8	ITGAV	CM 10	CM 13
S100A1_RYR2	S100A1	RYR2	CM 10	CM 13
TGM2_ITGB1	TGM2	ITGB1	CM 10	CM 13
TIMP1_CD63	TIMP1	CD63	CM 10	CM 13
CALM1_RYR2	CALM1	RYR2	CM 10	CM 14
COL1A2_CD36	COL1A2	CD36	CM 10	CM 14
COL1A2_ITGB1	COL1A2	ITGB1	CM 10	CM 14
COL3A1_ITGB1	COL3A1	ITGB1	CM 10	CM 14
COL4A1_ITGB1	COL4A1	ITGB1	CM 10	CM 14
COL6A1_ITGB1	COL6A1	ITGB1	CM 10	CM 14
COL6A2_ITGB1	COL6A2	ITGB1	CM 10	CM 14
COL6A3_ITGB1	COL6A3	ITGB1	CM 10	CM 14
FN1_ITGB1	FN1	ITGB1	CM 10	CM 14
LAMA2_ITGB1	LAMA2	ITGB1	CM 10	CM 14
LAMA2_RPSA	LAMA2	RPSA	CM 10	CM 14
LGALS1_ITGB1	LGALS1	ITGB1	CM 10	CM 14
LUM_ITGB1	LUM	ITGB1	CM 10	CM 14
S100A1_RYR2	S100A1	RYR2	CM 10	CM 14
TGM2_ITGB1	TGM2	ITGB1	CM 10	CM 14
TIMP1_CD63	TIMP1	CD63	CM 10	CM 14

Obstructive				
**Pair-Name**	**Ligand**	**Receptor**	**L_cell**	**R_cell**
LGALS1_PTPRC	LGALS1	PTPRC	CM 10	DC
TIMP1_CD63	TIMP1	CD63	CM 10	DC
TIMP1_CD63	TIMP1	CD63	CM 10	Leuko
LGALS1_ITGB1	LGALS1	ITGB1	CM 10	CM 4
LUM_ITGB1	LUM	ITGB1	CM 10	CM 4
S100A1_RYR2	S100A1	RYR2	CM 10	CM 4
TIMP1_CD63	TIMP1	CD63	CM 10	CM 4
CALM2_CACNA1C	CALM2	CACNA1C	CM 10	CM 5
CALM2_INSR	CALM2	INSR	CM 10	CM 5
CALM2_PDE1C	CALM2	PDE1C	CM 10	CM 5
LGALS1_ITGB1	LGALS1	ITGB1	CM 10	CM 5
LUM_ITGB1	LUM	ITGB1	CM 10	CM 5
S100A1_RYR2	S100A1	RYR2	CM 10	CM 5
TIMP1_CD63	TIMP1	CD63	CM 10	CM 5
LGALS1_ITGB1	LGALS1	ITGB1	CM 10	CM 8
LUM_ITGB1	LUM	ITGB1	CM 10	CM 8
S100A1_RYR2	S100A1	RYR2	CM 10	CM 8
TIMP1_CD63	TIMP1	CD63	CM 10	CM 8
TIMP1_CD63	TIMP1	CD63	CM 10	CM 10
TIMP1_CD63	TIMP1	CD63	CM 10	CM 12
CALM2_CACNA1C	CALM2	CACNA1C	CM 10	CM 13
CALM2_INSR	CALM2	INSR	CM 10	CM 13
CALM2_PDE1C	CALM2	PDE1C	CM 10	CM 13
LGALS1_ITGB1	LGALS1	ITGB1	CM 10	CM 13
LUM_ITGB1	LUM	ITGB1	CM 10	CM 13
S100A1_RYR2	S100A1	RYR2	CM 10	CM 13
TIMP1_CD63	TIMP1	CD63	CM 10	CM 13
TIMP1_CD63	TIMP1	CD63	CM 10	CM 14

Supplemental Figure S4. Reduced Cardiomyocyte Subtype Interactome Ligand-Receptor Pair Gene Expression in Obstructive vs. Nonobstructive HCM

Non-obstructive						*Obstructive*				
**Pair-Name**	**Ligand**	**Receptor**	**L_cell**	**R_cell**		**Pair-Name**	**Ligand**	**Receptor**	**L_cell**	**R_cell**
CALM1_CACNA1C	CALM1	CACNA1C	CM 9	CM 7		CALM1_INSR	CALM1	INSR	CM 9	CM 7
CALM1_PDE1C	CALM1	PDE1C	CM 9	CM 7		CALM1_PDE1C	CALM1	PDE1C	CM 9	CM 7
CALM1_RYR2	CALM1	RYR2	CM 9	CM 7		CALM1_RYR2	CALM1	RYR2	CM 9	CM 7
LAMA2_ITGB1	LAMA2	ITGB1	CM 9	CM 7		CALM2_INSR	CALM2	INSR	CM 9	CM 7
LGALS1_ITGB1	LGALS1	ITGB1	CM 9	CM 7		CALM2_PDE1C	CALM2	PDE1C	CM 9	CM 7
S100A1_RYR2	S100A1	RYR2	CM 9	CM 7		COL6A2_ITGB1	COL6A2	ITGB1	CM 9	CM 7
						LAMA2_ITGB1	LAMA2	ITGB1	CM 9	CM 7
CALM1_CACNA1C	CALM1	CACNA1C	CM 9	CM 9		LGALS1_ITGB1	LGALS1	ITGB1	CM 9	CM 7
CALM1_PDE1C	CALM1	PDE1C	CM 9	CM 9		LUM_ITGB1	LUM	ITGB1	CM 9	CM 7
CALM1_RYR2	CALM1	RYR2	CM 9	CM 9		S100A1_RYR2	S100A1	RYR2	CM 9	CM 7
LAMA2_ITGB1	LAMA2	ITGB1	CM 9	CM 9		SORBS1_INSR	SORBS1	INSR	CM 9	CM 7
LGALS1_ITGB1	LGALS1	ITGB1	CM 9	CM 9		TIMP1_CD63	TIMP1	CD63	CM 9	CM 7
S100A1_RYR2	S100A1	RYR2	CM 9	CM 9		VEGFA_ITGB1	VEGFA	ITGB1	CM 9	CM 7
CALM1_CACNA1C	CALM1	CACNA1C	CM 9	CM 13		CALM1_CACNA1C	CALM1	CACNA1C	CM 9	CM 9
CALM1_PDE1C	CALM1	PDE1C	CM 9	CM 13		CALM1_INSR	CALM1	INSR	CM 9	CM 9
CALM1_RYR2	CALM1	RYR2	CM 9	CM 13		CALM1_PDE1C	CALM1	PDE1C	CM 9	CM 9
LAMA2_ITGB1	LAMA2	ITGB1	CM 9	CM 13		CALM1_RYR2	CALM1	RYR2	CM 9	CM 9
LAMA2_RPSA	LAMA2	RPSA	CM 9	CM 13		CALM2_CACNA1C	CALM2	CACNA1C	CM 9	CM 9
LGALS1_ITGB1	LGALS1	ITGB1	CM 9	CM 13		CALM2_INSR	CALM2	INSR	CM 9	CM 9
MFGE8_ITGAV	MFGE8	ITGAV	CM 9	CM 13		CALM2_PDE1C	CALM2	PDE1C	CM 9	CM 9
S100A1_RYR2	S100A1	RYR2	CM 9	CM 13		COL6A2_ITGB1	COL6A2	ITGB1	CM 9	CM 9
						LAMA2_ITGB1	LAMA2	ITGB1	CM 9	CM 9
						LGALS1_ITGB1	LGALS1	ITGB1	CM 9	CM 9
						LUM_ITGB1	LUM	ITGB1	CM 9	CM 9
						S100A1_RYR2	S100A1	RYR2	CM 9	CM 9
						SORBS1_INSR	SORBS1	INSR	CM 9	CM 9
						TIMP1_CD63	TIMP1	CD63	CM 9	CM 9
						VEGFA_ITGB1	VEGFA	ITGB1	CM 9	CM 9
						VEGFA_NRP1	VEGFA	NRP1	CM 9	CM 9
						CALM1_CACNA1C	CALM1	CACNA1C	CM 9	CM 13
						CALM1_INSR	CALM1	INSR	CM 9	CM 13
						CALM1_PDE1C	CALM1	PDE1C	CM 9	CM 13
						CALM1_RYR2	CALM1	RYR2	CM 9	CM 13
						CALM2_CACNA1C	CALM2	CACNA1C	CM 9	CM 13
						CALM2_INSR	CALM2	INSR	CM 9	CM 13
						CALM2_PDE1C	CALM2	PDE1C	CM 9	CM 13
						COL6A2_ITGB1	COL6A2	ITGB1	CM 9	CM 13
						CYR61_CAV1	CYR61	CAV1	CM 9	CM 13
						LAMA2_ITGB1	LAMA2	ITGB1	CM 9	CM 13
						LGALS1_ITGB1	LGALS1	ITGB1	CM 9	CM 13
						LUM_ITGB1	LUM	ITGB1	CM 9	CM 13
						S100A1_RYR2	S100A1	RYR2	CM 9	CM 13
						SORBS1_INSR	SORBS1	INSR	CM 9	CM 13
						TIMP1_CD63	TIMP1	CD63	CM 9	CM 13
						VEGFA_ITGB1	VEGFA	ITGB1	CM 9	CM 13
						VEGFA_NRP1	VEGFA	NRP1	CM 9	CM 13

Supplemental Figure S5. Increased Cardiomyocyte Subtype Interactome Ligand-Receptor Pair Gene Expression in Obstructive vs. Nonobstructive HCM

Non-obstructive						Obstructive				
Pair-Name	Ligand	Receptor	L_cell	R_cell		Pair-Name	Ligand	Receptor	L_cell	R_cell
CALM1_CACNA1C	CALM1	CACNA1C	CM 10	CM 5		CALM2_CACNA1C	CALM2	CACNA1C	CM 10	CM 5
CALM1_INSR	CALM1	INSR	CM 10	CM 5		CALM2_INSR	CALM2	INSR	CM 10	CM 5
CALM1_PDE1C	CALM1	PDE1C	CM 10	CM 5		CALM2_PDE1C	CALM2	PDE1C	CM 10	CM 5
CALM1_RYR2	CALM1	RYR2	CM 10	CM 5		LGALS1_ITGB1	LGALS1	ITGB1	CM 10	CM 5
CALM2_CACNA1C	CALM2	CACNA1C	CM 10	CM 5		LUM_ITGB1	LUM	ITGB1	CM 10	CM 5
CALM2_INSR	CALM2	INSR	CM 10	CM 5		S100A1_RYR2	S100A1	RYR2	CM 10	CM 5
CALM2_PDE1C	CALM2	PDE1C	CM 10	CM 5		TIMP1_CD63	TIMP1	CD63	CM 10	CM 5
COL1A2_CD36	COL1A2	CD36	CM 10	CM 5		LGALS1_ITGB1	LGALS1	ITGB1	CM 10	CM 8
COL1A2_ITGAV	COL1A2	ITGAV	CM 10	CM 5		LUM_ITGB1	LUM	ITGB1	CM 10	CM 8
COL1A2_ITGB1	COL1A2	ITGB1	CM 10	CM 5		S100A1_RYR2	S100A1	RYR2	CM 10	CM 8
COL3A1_ITGB1	COL3A1	ITGB1	CM 10	CM 5		TIMP1_CD63	TIMP1	CD63	CM 10	CM 8
COL4A1_ITGAV	COL4A1	ITGAV	CM 10	CM 5		TIMP1_CD63	TIMP1	CD63	CM 10	CM 10
COL4A1_ITGB1	COL4A1	ITGB1	CM 10	CM 5		TIMP1_CD63	TIMP1	CD63	CM 10	CM 12
COL6A1_ITGB1	COL6A1	ITGB1	CM 10	CM 5		CALM2_CACNA1C	CALM2	CACNA1C	CM 10	CM 13
COL6A2_ITGB1	COL6A2	ITGB1	CM 10	CM 5		CALM2_INSR	CALM2	INSR	CM 10	CM 13
COL6A3_ITGB1	COL6A3	ITGB1	CM 10	CM 5		CALM2_PDE1C	CALM2	PDE1C	CM 10	CM 13
FN1_ITGAV	FN1	ITGAV	CM 10	CM 5		LGALS1_ITGB1	LGALS1	ITGB1	CM 10	CM 13
FN1_ITGB1	FN1	ITGB1	CM 10	CM 5		LUM_ITGB1	LUM	ITGB1	CM 10	CM 13
LAMA2_ITGA7	LAMA2	ITGA7	CM 10	CM 5		S100A1_RYR2	S100A1	RYR2	CM 10	CM 13
LAMA2_ITGB1	LAMA2	ITGB1	CM 10	CM 5		TIMP1_CD63	TIMP1	CD63	CM 10	CM 13
LAMA2_RPSA	LAMA2	RPSA	CM 10	CM 5		TIMP1_CD63	TIMP1	CD63	CM 10	CM 14
LGALS1_ITGB1	LGALS1	ITGB1	CM 10	CM 5		TIMP1_CD63	TIMP1	CD63	CM 10	Fibro 1
LUM_ITGB1	LUM	ITGB1	CM 10	CM 5		TIMP1_CD63	TIMP1	CD63	CM 10	Fibro 4
MFGE8_ITGAV	MFGE8	ITGAV	CM 10	CM 5		TIMP1_CD63	TIMP1	CD63	CM 10	Fibro 5
S100A1_RYR2	S100A1	RYR2	CM 10	CM 5						
SORBS1_INSR	SORBS1	INSR	CM 10	CM 5						
TGM2_ITGB1	TGM2	ITGB1	CM 10	CM 5						
TIMP1_CD63	TIMP1	CD63	CM 10	CM 5						
CALM1_CACNA1C	CALM1	CACNA1C	CM 10	CM 8						
CALM1_PDE1C	CALM1	PDE1C	CM 10	CM 8						
CALM1_RYR2	CALM1	RYR2	CM 10	CM 8						
CALM2_CACNA1C	CALM2	CACNA1C	CM 10	CM 8						
CALM2_PDE1C	CALM2	PDE1C	CM 10	CM 8						
COL1A2_CD36	COL1A2	CD36	CM 10	CM 8						
COL1A2_ITGB1	COL1A2	ITGB1	CM 10	CM 8						
COL3A1_ITGB1	COL3A1	ITGB1	CM 10	CM 8						
COL4A1_ITGB1	COL4A1	ITGB1	CM 10	CM 8						
COL6A1_ITGB1	COL6A1	ITGB1	CM 10	CM 8						
COL6A2_ITGB1	COL6A2	ITGB1	CM 10	CM 8						
COL6A3_ITGB1	COL6A3	ITGB1	CM 10	CM 8						
FN1_ITGB1	FN1	ITGB1	CM 10	CM 8						
LAMA2_ITGB1	LAMA2	ITGB1	CM 10	CM 8						
LGALS1_ITGB1	LGALS1	ITGB1	CM 10	CM 8						
LUM_ITGB1	LUM	ITGB1	CM 10	CM 8						
S100A1_RYR2	S100A1	RYR2	CM 10	CM 8						
TGM2_ITGB1	TGM2	ITGB1	CM 10	CM 8						
TIMP1_CD63	TIMP1	CD63	CM 10	CM 8						
CALM1_RYR2	CALM1	RYR2	CM 10	CM 10						
COL1A2_CD36	COL1A2	CD36	CM 10	CM 10						
COL1A2_ITGB1	COL1A2	ITGB1	CM 10	CM 10						
COL3A1_ITGB1	COL3A1	ITGB1	CM 10	CM 10						
COL4A1_ITGB1	COL4A1	ITGB1	CM 10	CM 10						
COL6A1_ITGB1	COL6A1	ITGB1	CM 10	CM 10						
COL6A2_ITGB1	COL6A2	ITGB1	CM 10	CM 10						
COL6A3_ITGB1	COL6A3	ITGB1	CM 10	CM 10						
FN1_ITGB1	FN1	ITGB1	CM 10	CM 10						
LAMA2_ITGB1	LAMA2	ITGB1	CM 10	CM 10						
LGALS1_ITGB1	LGALS1	ITGB1	CM 10	CM 10						
LUM_ITGB1	LUM	ITGB1	CM 10	CM 10						
S100A1_RYR2	S100A1	RYR2	CM 10	CM 10						
TGM2_ITGB1	TGM2	ITGB1	CM 10	CM 10						
TIMP1_CD63	TIMP1	CD63	CM 10	CM 10						
CALM1_RYR2	CALM1	RYR2	CM 10	CM 12						
COL1A2_CD36	COL1A2	CD36	CM 10	CM 12						
COL1A2_ITGB1	COL1A2	ITGB1	CM 10	CM 12						
COL3A1_ITGB1	COL3A1	ITGB1	CM 10	CM 12						
COL4A1_ITGB1	COL4A1	ITGB1	CM 10	CM 12						
COL6A1_ITGB1	COL6A1	ITGB1	CM 10	CM 12						
COL6A2_ITGB1	COL6A2	ITGB1	CM 10	CM 12						
COL6A3_ITGB1	COL6A3	ITGB1	CM 10	CM 12						
FN1_ITGB1	FN1	ITGB1	CM 10	CM 12						
LAMA2_ITGB1	LAMA2	ITGB1	CM 10	CM 12						
LGALS1_ITGB1	LGALS1	ITGB1	CM 10	CM 12						
LUM_ITGB1	LUM	ITGB1	CM 10	CM 12						
S100A1_RYR2	S100A1	RYR2	CM 10	CM 12						
TGM2_ITGB1	TGM2	ITGB1	CM 10	CM 12						
TIMP1_CD63	TIMP1	CD63	CM 10	CM 12						
CALM1_CACNA1C	CALM1	CACNA1C	CM 10	CM 13						
CALM1_PDE1C	CALM1	PDE1C	CM 10	CM 13						
CALM1_RYR2	CALM1	RYR2	CM 10	CM 13						
CALM2_CACNA1C	CALM2	CACNA1C	CM 10	CM 13						
CALM2_PDE1C	CALM2	PDE1C	CM 10	CM 13						
COL1A2_CD36	COL1A2	CD36	CM 10	CM 13						
COL1A2_ITGAV	COL1A2	ITGAV	CM 10	CM 13						
COL1A2_ITGB1	COL1A2	ITGB1	CM 10	CM 13						
COL3A1_ITGB1	COL3A1	ITGB1	CM 10	CM 13						
COL4A1_ITGAV	COL4A1	ITGAV	CM 10	CM 13						
COL4A1_ITGB1	COL4A1	ITGB1	CM 10	CM 13						
COL6A1_ITGB1	COL6A1	ITGB1	CM 10	CM 13						
COL6A2_ITGB1	COL6A2	ITGB1	CM 10	CM 13						
COL6A3_ITGB1	COL6A3	ITGB1	CM 10	CM 13						
FN1_ITGAV	FN1	ITGAV	CM 10	CM 13						
FN1_ITGB1	FN1	ITGB1	CM 10	CM 13						
LAMA2_ITGB1	LAMA2	ITGB1	CM 10	CM 13						
LAMA2_RPSA	LAMA2	RPSA	CM 10	CM 13						
LGALS1_ITGB1	LGALS1	ITGB1	CM 10	CM 13						
LUM_ITGB1	LUM	ITGB1	CM 10	CM 13						
MFGE8_ITGAV	MFGE8	ITGAV	CM 10	CM 13						
S100A1_RYR2	S100A1	RYR2	CM 10	CM 13						
TGM2_ITGB1	TGM2	ITGB1	CM 10	CM 13						
TIMP1_CD63	TIMP1	CD63	CM 10	CM 13						
CALM1_RYR2	CALM1	RYR2	CM 10	CM 14						
COL1A2_CD36	COL1A2	CD36	CM 10	CM 14						
COL1A2_ITGB1	COL1A2	ITGB1	CM 10	CM 14						
COL3A1_ITGB1	COL3A1	ITGB1	CM 10	CM 14						
COL4A1_ITGB1	COL4A1	ITGB1	CM 10	CM 14						
COL6A1_ITGB1	COL6A1	ITGB1	CM 10	CM 14						
COL6A2_ITGB1	COL6A2	ITGB1	CM 10	CM 14						
COL6A3_ITGB1	COL6A3	ITGB1	CM 10	CM 14						
FN1_ITGB1	FN1	ITGB1	CM 10	CM 14						
LAMA2_ITGB1	LAMA2	ITGB1	CM 10	CM 14						
LAMA2_RPSA	LAMA2	RPSA	CM 10	CM 14						
LGALS1_ITGB1	LGALS1	ITGB1	CM 10	CM 14						
LUM_ITGB1	LUM	ITGB1	CM 10	CM 14						
S100A1_RYR2	S100A1	RYR2	CM 10	CM 14						
TGM2_ITGB1	TGM2	ITGB1	CM 10	CM 14						
TIMP1_CD63	TIMP1	CD63	CM 10	CM 14						
CALM1_RYR2	CALM1	RYR2	CM 10	Fibro 1						
COL1A2_CD36	COL1A2	CD36	CM 10	Fibro 1						
COL1A2_ITGB1	COL1A2	ITGB1	CM 10	Fibro 1						
COL3A1_ITGB1	COL3A1	ITGB1	CM 10	Fibro 1						
COL4A1_ITGB1	COL4A1	ITGB1	CM 10	Fibro 1						
COL6A1_ITGB1	COL6A1	ITGB1	CM 10	Fibro 1						
COL6A2_ITGB1	COL6A2	ITGB1	CM 10	Fibro 1						
COL6A3_ITGB1	COL6A3	ITGB1	CM 10	Fibro 1						
FN1_ITGB1	FN1	ITGB1	CM 10	Fibro 1						
LAMA2_ITGB1	LAMA2	ITGB1	CM 10	Fibro 1						
LGALS1_ITGB1	LGALS1	ITGB1	CM 10	Fibro 1						
LUM_ITGB1	LUM	ITGB1	CM 10	Fibro 1						
S100A1_RYR2	S100A1	RYR2	CM 10	Fibro 1						
TGM2_ITGB1	TGM2	ITGB1	CM 10	Fibro 1						
TIMP1_CD63	TIMP1	CD63	CM 10	Fibro 1						
CALM1_RYR2	CALM1	RYR2	CM 10	Fibro 4						
COL1A2_CD36	COL1A2	CD36	CM 10	Fibro 4						
COL1A2_ITGB1	COL1A2	ITGB1	CM 10	Fibro 4						
COL3A1_ITGB1	COL3A1	ITGB1	CM 10	Fibro 4						
COL4A1_ITGB1	COL4A1	ITGB1	CM 10	Fibro 4						
COL6A1_ITGB1	COL6A1	ITGB1	CM 10	Fibro 4						
COL6A2_ITGB1	COL6A2	ITGB1	CM 10	Fibro 4						
COL6A3_ITGB1	COL6A3	ITGB1	CM 10	Fibro 4						
FN1_ITGB1	FN1	ITGB1	CM 10	Fibro 4						
LAMA2_ITGB1	LAMA2	ITGB1	CM 10	Fibro 4						
LGALS1_ITGB1	LGALS1	ITGB1	CM 10	Fibro 4						
LUM_ITGB1	LUM	ITGB1	CM 10	Fibro 4						
S100A1_RYR2	S100A1	RYR2	CM 10	Fibro 4						
TGM2_ITGB1	TGM2	ITGB1	CM 10	Fibro 4						
TIMP1_CD63	TIMP1	CD63	CM 10	Fibro 4						
CALM1_RYR2	CALM1	RYR2	CM 10	Fibro 5						
COL1A2_CD36	COL1A2	CD36	CM 10	Fibro 5						
COL1A2_ITGB1	COL1A2	ITGB1	CM 10	Fibro 5						
COL3A1_ITGB1	COL3A1	ITGB1	CM 10	Fibro 5						
COL4A1_ITGB1	COL4A1	ITGB1	CM 10	Fibro 5						
COL6A1_ITGB1	COL6A1	ITGB1	CM 10	Fibro 5						
COL6A2_ITGB1	COL6A2	ITGB1	CM 10	Fibro 5						
COL6A3_ITGB1	COL6A3	ITGB1	CM 10	Fibro 5						
FN1_ITGB1	FN1	ITGB1	CM 10	Fibro 5						
LAMA2_ITGB1	LAMA2	ITGB1	CM 10	Fibro 5						
LAMA2_RPSA	LAMA2	RPSA	CM 10	Fibro 5						
LGALS1_ITGB1	LGALS1	ITGB1	CM 10	Fibro 5						
LUM_ITGB1	LUM	ITGB1	CM 10	Fibro 5						
S100A1_RYR2	S100A1	RYR2	CM 10	Fibro 5						
TGM2_ITGB1	TGM2	ITGB1	CM 10	Fibro 5						
TIMP1_CD63	TIMP1	CD63	CM 10	Fibro 5						

Supplemental Figure S6. Reduced Cardiomyocyte-Fibroblast Subtype Interactome Ligand-Receptor Pair Gene Expression in Obstructive vs. Nonobstructive HCM

Non-obstructive				
**Pair-Name**	**Ligand**	**Receptor**	**L_cell**	**R_cell**
CALM1_RYR2	CALM1	RYR2	Fibro 2	Fibro 2
COL1A2_CD36	COL1A2	CD36	Fibro 2	Fibro 2
COL1A2_ITGB1	COL1A2	ITGB1	Fibro 2	Fibro 2
COL3A1_ITGB1	COL3A1	ITGB1	Fibro 2	Fibro 2
COL4A1_ITGB1	COL4A1	ITGB1	Fibro 2	Fibro 2
COL6A1_ITGB1	COL6A1	ITGB1	Fibro 2	Fibro 2
COL6A2_ITGB1	COL6A2	ITGB1	Fibro 2	Fibro 2
COL6A3_ITGB1	COL6A3	ITGB1	Fibro 2	Fibro 2
FN1_ITGB1	FN1	ITGB1	Fibro 2	Fibro 2
LAMA2_ITGB1	LAMA2	ITGB1	Fibro 2	Fibro 2
LGALS1_ITGB1	LGALS1	ITGB1	Fibro 2	Fibro 2
LUM_ITGB1	LUM	ITGB1	Fibro 2	Fibro 2
S100A1_RYR2	S100A1	RYR2	Fibro 2	Fibro 2
TIMP1_CD63	TIMP1	CD63	Fibro 2	Fibro 2
TIMP2_ITGB1	TIMP2	ITGB1	Fibro 2	Fibro 2
VCAN_ITGB1	VCAN	ITGB1	Fibro 2	Fibro 2
CALM1_CACNA1C	CALM1	CACNA1C	CM 9	CM 9
CALM1_PDE1C	CALM1	PDE1C	CM 9	CM 9
CALM1_RYR2	CALM1	RYR2	CM 9	CM 9
LAMA2_ITGB1	LAMA2	ITGB1	CM 9	CM 9
LGALS1_ITGB1	LGALS1	ITGB1	CM 9	CM 9
S100A1_RYR2	S100A1	RYR2	CM 9	CM 9

Obstructive				
**Pair-Name**	**Ligand**	**Receptor**	**L_cell**	**R_cell**
TIMP1_CD63	TIMP1	CD63	Fibro 2	Fibro 1
APP_LRP1	APP	LRP1	Fibro 2	Fibro 2
COL1A1_CD36	COL1A1	CD36	Fibro 2	Fibro 2
COL1A1_ITGB1	COL1A1	ITGB1	Fibro 2	Fibro 2
COL1A2_CD36	COL1A2	CD36	Fibro 2	Fibro 2
COL1A2_ITGB1	COL1A2	ITGB1	Fibro 2	Fibro 2
COL3A1_ITGB1	COL3A1	ITGB1	Fibro 2	Fibro 2
COL6A1_ITGB1	COL6A1	ITGB1	Fibro 2	Fibro 2
COL6A2_ITGB1	COL6A2	ITGB1	Fibro 2	Fibro 2
COL6A3_ITGB1	COL6A3	ITGB1	Fibro 2	Fibro 2
CTGF_LRP1	CTGF	LRP1	Fibro 2	Fibro 2
FBLN1_ITGB1	FBLN1	ITGB1	Fibro 2	Fibro 2
FBN1_ITGB1	FBN1	ITGB1	Fibro 2	Fibro 2
FN1_ITGB1	FN1	ITGB1	Fibro 2	Fibro 2
FN1_SDC2	FN1	SDC2	Fibro 2	Fibro 2
HSP90AA1_LRP1	HSP90AA1	LRP1	Fibro 2	Fibro 2
HSPG2_ITGB1	HSPG2	ITGB1	Fibro 2	Fibro 2
HSPG2_LRP1	HSPG2	LRP1	Fibro 2	Fibro 2
LAMA2_ITGB1	LAMA2	ITGB1	Fibro 2	Fibro 2
LAMC1_ITGB1	LAMC1	ITGB1	Fibro 2	Fibro 2
LGALS1_ITGB1	LGALS1	ITGB1	Fibro 2	Fibro 2
LGALS3BP_ITGB1	LGALS3BP	ITGB1	Fibro 2	Fibro 2
LUM_ITGB1	LUM	ITGB1	Fibro 2	Fibro 2
MMP2_SDC2	MMP2	SDC2	Fibro 2	Fibro 2
PSAP_LRP1	PSAP	LRP1	Fibro 2	Fibro 2
SERPINE2_LRP1	SERPINE2	LRP1	Fibro 2	Fibro 2
SERPING1_LRP1	SERPING1	LRP1	Fibro 2	Fibro 2
TFPI_LRP1	TFPI	LRP1	Fibro 2	Fibro 2
TIMP1_CD63	TIMP1	CD63	Fibro 2	Fibro 2
TIMP2_ITGB1	TIMP2	ITGB1	Fibro 2	Fibro 2
VCAN_ITGB1	VCAN	ITGB1	Fibro 2	Fibro 2
CALM1_CACNA1C	CALM1	CACNA1C	CM 9	CM 9
CALM1_INSR	CALM1	INSR	CM 9	CM 9
CALM1_PDE1C	CALM1	PDE1C	CM 9	CM 9
CALM1_RYR2	CALM1	RYR2	CM 9	CM 9
CALM2_CACNA1C	CALM2	CACNA1C	CM 9	CM 9
CALM2_INSR	CALM2	INSR	CM 9	CM 9
CALM2_PDE1C	CALM2	PDE1C	CM 9	CM 9
COL6A2_ITGB1	COL6A2	ITGB1	CM 9	CM 9
LAMA2_ITGB1	LAMA2	ITGB1	CM 9	CM 9
LGALS1_ITGB1	LGALS1	ITGB1	CM 9	CM 9
LUM_ITGB1	LUM	ITGB1	CM 9	CM 9
S100A1_RYR2	S100A1	RYR2	CM 9	CM 9
SORBS1_INSR	SORBS1	INSR	CM 9	CM 9
TIMP1_CD63	TIMP1	CD63	CM 9	CM 9
VEGFA_ITGB1	VEGFA	ITGB1	CM 9	CM 9
VEGFA_NRP1	VEGFA	NRP1	CM 9	CM 9

Supplemental Figure S7. Increased Cardiomyocyte-Fibroblast Subtype Interactome Ligand-Receptor Pair Gene Expression in Obstructive vs. Nonobstructive HCM
